# Correlating phylogenetic and functional diversity of the *nod*-free but nodulating *Bradyrhizobium* phylogroup

**DOI:** 10.1093/ismejo/wraf030

**Published:** 2025-02-17

**Authors:** Lu Ling, Alicia Camuel, Sishuo Wang, Xiaojun Wang, Tianhua Liao, Jinjin Tao, Xingqin Lin, Nico Nouwen, Eric Giraud, Haiwei Luo

**Affiliations:** Simon F. S. Li Marine Science Laboratory, School of Life Sciences and State Key Laboratory of Agrobiotechnology, The Chinese University of Hong Kong, Shatin, Hong Kong SAR, 999077, China; Shenzhen Research Institute, The Chinese University of Hong Kong, Shenzhen, 518000, China; IRD, Laboratoire des Symbioses Tropicales et Méditerranéennes (LSTM), UMR IRD/Institut Agro/INRAE/Université de Montpellier/CIRAD, TA-A82/J-Campus de Baillarguet 34398, Montpellier Cedex 5, France; PHIM Plant Health Institute, Universite de Montpellier, IRD, CIRAD, INRAE, Institut Agro, Montpellier, France; Simon F. S. Li Marine Science Laboratory, School of Life Sciences and State Key Laboratory of Agrobiotechnology, The Chinese University of Hong Kong, Shatin, Hong Kong SAR, 999077, China; Shenzhen Research Institute, The Chinese University of Hong Kong, Shenzhen, 518000, China; Simon F. S. Li Marine Science Laboratory, School of Life Sciences and State Key Laboratory of Agrobiotechnology, The Chinese University of Hong Kong, Shatin, Hong Kong SAR, 999077, China; Simon F. S. Li Marine Science Laboratory, School of Life Sciences and State Key Laboratory of Agrobiotechnology, The Chinese University of Hong Kong, Shatin, Hong Kong SAR, 999077, China; Shenzhen Research Institute, The Chinese University of Hong Kong, Shenzhen, 518000, China; IRD, Laboratoire des Symbioses Tropicales et Méditerranéennes (LSTM), UMR IRD/Institut Agro/INRAE/Université de Montpellier/CIRAD, TA-A82/J-Campus de Baillarguet 34398, Montpellier Cedex 5, France; PHIM Plant Health Institute, Universite de Montpellier, IRD, CIRAD, INRAE, Institut Agro, Montpellier, France; IRD, Laboratoire des Symbioses Tropicales et Méditerranéennes (LSTM), UMR IRD/Institut Agro/INRAE/Université de Montpellier/CIRAD, TA-A82/J-Campus de Baillarguet 34398, Montpellier Cedex 5, France; PHIM Plant Health Institute, Universite de Montpellier, IRD, CIRAD, INRAE, Institut Agro, Montpellier, France; Simon F. S. Li Marine Science Laboratory, School of Life Sciences and State Key Laboratory of Agrobiotechnology, The Chinese University of Hong Kong, Shatin, Hong Kong SAR, 999077, China; Institute of Environment, Energy and Sustainability, The Chinese University of Hong Kong, Shatin, Hong Kong SAR, 999077, China

**Keywords:** *Bradyrhizobium*, *Aeschynomene*, nodulation, phylogenetic diversity, population genomics

## Abstract

*Bradyrhizobium* is a main rhizobial lineage of which most members nodulate legume plants using Nod factors synthetized by the *nod* genes. However, members of the Photosynthetic supergroup (phylogroup) within *Bradyrhizobium* are *nod*-free, but still capable of establishing nitrogen-fixing nodules with some tropical legumes of the *Aeschynomene* genus. These unusual findings are based on the genomic sequences of only 13 Photosynthetic *Bradyrhizobium* strains, and almost all were isolated from *Aeschynomene* nodules. Here, we report that Photosynthetic *Bradyrhizobium* supergroup members are more abundantly associated with rice root (endosphere and rhizosphere) compared to grassland, forest, and maize samples based on *rpoB* amplicon sequence analyses. We sequenced 263 new isolates of this supergroup mostly from two main subspecies of cultivated rice (*Oryza sativa* L. spp. *indica* and *japonica*). The extended supergroup comprises three major clades with their diversity broadly covering the natural community of this supergroup: a basal clade with significant expansion of its diversity, a clade composed by two phylogenetically diverse strains including one newly isolated, and a new clade exclusively represented by our new strains. Although this supergroup members universally lack the canonical *nod* genes, all 28 assayed strains covering the broad diversity induced nodules on *Aeschynomene indica*. The three clades displayed important differences in the efficiency of symbiosis, aligning well with their phylogenetic divergence. With this expanded ecological, phylogenetic, and functional diversity, we conclude that the *nod* factor-independent nodulation of *Aeschynomene* is a common trait of this supergroup, in contrast to the photosynthetic trait originally thought of as its unifying feature.

## Introduction

The genus *Bradyrhizobium* is one of the largest and most diverse rhizobial genera and the primary symbiont of many legumes [1, 2]. It encompasses seven phylogenetic supergroups (phylogroups) [3, 4]. The Photosynthetic *Bradyrhizobium* (PB) supergroup is one of the most special as photosynthesis is a rare trait in rhizobia [3]. Members belonging to this supergroup engage in a mutualistic relationship with some tropical semi-aquatic species of the *Aeschynomene* genus [5], and they form nodules not only on roots but also on stems [6, 7]. Furthermore, none of the completely sequenced PB members carry *nodABC* genes encoding the enzymes synthesizing the core structure of the nodulation factors (NFs). The only exception is *Bradyrhizobium* sp. ORS285, which can use both a *nod*-dependent and -independent pathway reliant on the *Aeschynomene* host plant [8, 9]. This indicates that the PB phylogroup uses a new mechanism to associate with legume hosts that differs from the traditional universal NF-based pathway [10, 11]. Moreover, all known PB strains carry photosynthetic genes except for four deep-branching members isolated from French Guiana as represented by *Bradyrhizobium* sp. STM3843 [7, 12].

Despite these unique features, research on PB has been limited. Currently, there are 13 PB strains that have their genomes sequenced, largely limiting genome-based analysis on this important *Bradyrhizobium* phylogroup. Although most PB strains were isolated from *Aeschynomene* spp. nodules, the PB phylogroup was also detected in paddy soil, rice roots as well as in lake water [13–16], suggesting that its members have much wider ecological habitats than previously thought. Here, we report that rice fields are an important reservoir of PB among the terrestrial ecosystems through *rpoB* amplicon sequencing analysis. We isolated and genome-sequenced 263 isolates predominantly from three habitats (endosphere, rhizosphere, bulk soil) of two main subspecies of cultivated rice (*Oryza sativa* subsp. *indica* and *japonica*) sampled from two distant locations and discovered a new, deep-branching clade. We further show that the phylogenomic diversity of PB members match well with their symbiotic efficiency with *Aeschynomene indica*.

## Materials and methods

All the methodological details are described in the Supplementary Text.

### Sample collection, processing, and bacterial isolation

We collected various non-legume plants from sites with important differences in precipitation and temperature. These included rice (*O. sativa* subsp. *indica*) from Hunan province (27.948°N, 113.221°E), rice (*O. sativa* subsp. *japonica*) from Hong Kong, China (22.418°N, 114.080°E), maize (*Zea mays*) from Anhui province (33.385°N, 117.255°E) and Shanxi province (36.568°N, 111.876°E), *Houttuynia cordata* from Hunan grassland (27.943°N, 113.225°E), and *Camphora officinarum* from Hunan forest (27.944°N, 113.226°E). Three typical habitats (bulk soil, rhizosphere, and endosphere) of each plant species (three replicates, 5–10 m apart) were used for *Bradyrhizobium* isolation using a modified arabinose-gluconate medium and *rpoB* amplicon sequencing. Basic soil characteristics (e.g. soil water content, pH, soil organic carbon) were measured. The taxonomic affiliation of isolates was determined by 16S rRNA gene analysis. We also collected banana (*Musa*), chili (*Capsicum*), lettuce (*Lactuca*), white radish (*Raphanus*), white gourd (*Benincasa*), and Mexican ageratum (*Ageratum houstonianum*), but *Bradyrhizobium* isolation from these samples had limited success.

### Amplicon sequencing and analysis

DNA was extracted from fresh soil and plant root samples (0.25 g) using the DNeasy PowerSoil Pro Kit (QIAGEN) according to the manufacturer’s protocol and then sent to the company (Magigene, Guangdong) for *rpoB* amplicon sequencing. As the quality and/or quantity of DNA extracted from a few samples, including the Hong Kong rice roots, one replicate of the Hunan rice root, the roots and rhizosphere soils of *Houttuynia cordata* and *Camphora officinarum*, and the Shanxi maize roots, were poor, *rpoB* amplicon sequencing was not performed on these samples.

The quality control of the raw reads was performed with Trimmomatic v0.39 [17]. Subsequently, the paired-end reads of the *rpoB* amplicon sequences were processed with a denoising algorithm (DADA2) [18] implemented in QIIME2 [19] to perform sequence denoising, dereplication, and chimera filtering to generate amplicon sequence variants (ASVs). The generated ASVs were filtered out the non-*rpoB* sequences and then assigned to each *Bradyrhizobium* supergroup through a phylogenetic placement method [20], which was used to determine their relative abundance. Meanwhile, the ASVs classified into the Photosynthetic *Bradyrhizobium* supergroup were further assigned to each of the three clades.

To distinguish each *Bradyrhizobium* supergroup, two *rpoB* gene trees, using the full length (Fig. S1A) and amplified region (Fig. S1B) respectively, were constructed with IQ-Tree v2.2.0 [21] based on the sequence of *rpoB* genes retrieved from *Bradyrhizobium* genomes (263 new genomes and 566 public genomes downloaded from the NCBI GenBank database). To identify the diversity of Photosynthetic *Bradyrhizobium* in each sample, a *rpoB* gene tree was also built by combining ASVs and amplified regions from Photosynthetic *Bradyrhizobium* genomes (263 new genomes and 13 public genomes). Although phylogenetic resolution of the *rpoB* gene faded when the short amplified region was used (Fig. S1B), members from each supergroup remain clustered though broken into several subclades, suggesting the use of the tree based on short amplified region has limited effect on ASVs assignment using the commonly used phylogenetic placement method [22].

### Genome sequencing and analysis

Bacteria Genomic DNA Extraction Kit [OMEGA Bacterial DNA Kit D3350] was used to extract genomic DNA from each of the 263 isolates. NanoDrop™ 2000 [Thermo Fisher, USA] was used to assess the quality of the extracted DNA samples with the following criteria, A260/A280 > 1.8, A260/A230 > 2.0, and A260/A270 > 1.0. Whole genome sequencing was performed in Wuhan Huada Gene Biotechnology Company by using the MGISEQ-2000 PE150 + 150 + 10 + 10 platform (paired-end reads of 150 bp).

A phylogenomic tree of *Bradyrhizobium* (Fig. S2) was built using IQ-Tree v2.2.0 [21] with our 263 isolates and 566 public genomes (outgroup included) based on 123 shared single-copy genes identified in a previous phylogenomic study of *Bradyrhizobium* [23]. As our isolates mainly fall into Photosynthetic *Bradyrhizobium* supergroup, we also performed phylogenomic and comparative genomic analyses for this supergroup to understand their phylogenetic and population structure. PopCOGenT [24] was used to delineate genetically isolated populations (reported as main clusters or MCs) for the 276 Photosynthetic *Bradyrhizobium* genomes (13 public genomes and 263 new genomes). The main cluster boundaries delineated by this method are based on recent gene flow barriers, matching well with the idea that bacterial speciation often proceeds rapidly [24].

### Physiological assays

We selected 28 strains spanning over the main clades and the main clusters defined by PopCOGenT for symbiotic assays including nodulation and nitrogen fixation capabilities on the tropical legume species *A. indica*. Additionally, the nitrogenase enzyme activity under free-living conditions and methanol utilization were also detected.

## Results and discussion

### Photosynthetic supergroup of *Bradyrhizobium* is enriched in rice cropland

To distinguish *Bradyrhizobium* supergroup, the *rpoB* gene tree (Fig. S1A) has been constructed in addition to the phylogenomic tree (Fig. S2). *Bradyrhizobium* members were grouped into seven supergroups including the PB in these two trees. Their largely congruent tree topological structures support the idea that *rpoB* is an appropriate gene marker [25] to distinguish *Bradyrhizobium* supergroups. We therefore designed specific *rpoB* primers to investigate the relative abundance and diversity of each *Bradyrhizobium* supergroup in different terrestrial ecosystems including five plant species (at least one plant species specific to each ecosystem type was chosen: *O. sativa* subsp. *indica*, *O. sativa* subsp. *japonica*, and *Zea mays* for cropland, *Houttuynia cordata* for grassland, *Camphora officinarum* for forest).

**Figure 1:**
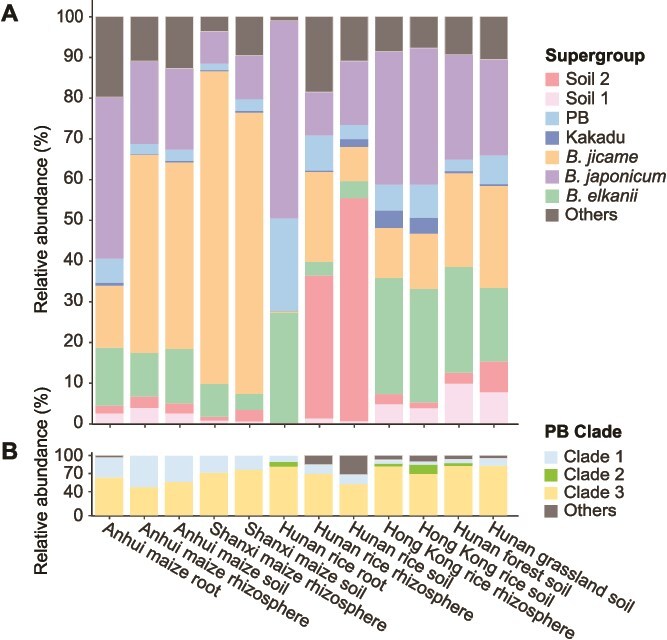
The relative abundance of each *Bradyrhizobium* supergroup (A) and each major clade within the Photosynthetic supergroup of *Bradyrhizobium* (B). The relative abundance of each *Bradyrhizobium* supergroup / PB clade was determined by dividing the number of reads assigned to each supergroup / clade by the total number of filtered reads assigned to *Bradyrhizobium* / PB. Abbreviations: *B. elkanii*, *B. jicamae*, and *B. japonicum* represent *Bradyrhizobium elkanii*, *Bradyrhizobium jicamae*, and *Bradyrhizobium japonicum* supergroups, respectively.

We show that most *Bradyrhizobium* supergroups, including PB, are widely distributed in cropland, forest, and grassland (Fig. 1A, Dataset S1). The relative abundance of each *Bradyrhizobium* supergroup varies across the ecosystems. PB (especially Clade 3) is enriched in the root endosphere and rhizosphere of rice (Fig. 1A and B, Datasets S1 and S2), suggesting that members of this supergroup have the potential to act as plant growth promoting bacteria (PGPB) to promote rice growth and yield. This possibility is supported by a previous report that PB strains are able to heavily colonize rice roots, invade the tissues to become rice endophytic strains, and significantly promote rice growth [13, 15]. We show that the relative abundance of PB supergroup is significantly negatively correlated with the amount of NH_4_^+^ present in the soil (Fig. S3), indicating that they may be more competitive and abundant in NH_4_^+^-deficient conditions.

### Our culture collection contributes a new, deep-branching clade of the Photosynthetic supergroup of Bradyrhizobium

**Figure 2:**
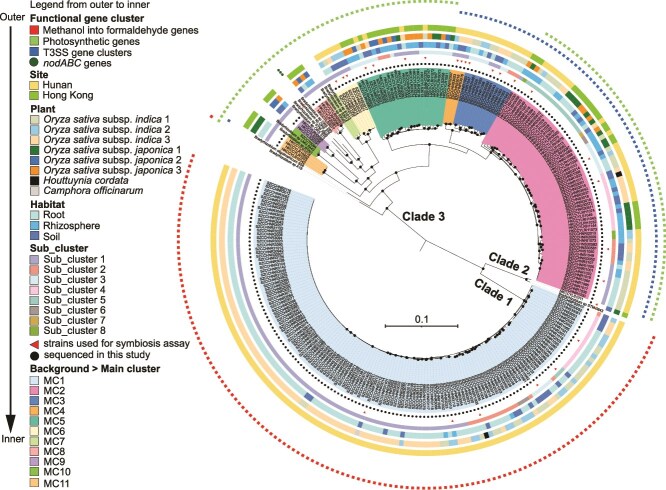
The phylogenomic tree and population delineation of the “photosynthetic” *Bradyrhizobium* (PB) supergroup. The phylogenomic tree was rooted using the minimum variance (MV) method. The 263 PB strains sequenced in the present study were indicated by black dots in the innermost layer surrounding the phylogeny. The ultrafast bootstrap values higher than or equal to 95% calculated by IQ-tree were labeled on the nodes with black circles. The phylogenomic tree of the Photosynthetic *Bradyrhizobium* based on the minimal ancestor deviation (MAD) rooting method was displayed in Fig. S4. The scale bar indicates the number of substitutions per site.

Of the 263 new PB supergroup strains, 205 were collected from three *O. sativa* subsp. *indica* plants sampled from a rice field in Hunan, and 55 were isolated from three *O. sativa* subsp. *japonica* plants sampled from a rice field in Hong Kong. The remaining three strains were isolated from grassland and forest soils. With genome sequences of these new isolates (Dataset S3) and 13 known isolates, the genome-based phylogeny of the PB supergroup is split into three deep-branching clades (see Fig. S2 for their phylogenetic position in the species tree of the entire *Bradyrhizobium* genus). Clade 3 represents the evolutionarily basal lineage. It initially comprised only 12 strains (Dataset S4), but we here contributed 122 new strains representing several new lineages delineated as distinct genetically isolated main clusters (Fig. 2, Datasets S3 and S4; also discussed in the next section). Clade 2 has only two strains (the publicly available *Bradyrhizobium* sp. STM3843 and a new strain HKCCYLS1011 isolated from *O. sativa* subsp. *japonica*) and represents a sister clade to Clade 1. Clade 1 consists of 140 newly isolated strains (Fig. 2). The evolutionary branching order of the three clades is verified with two outgroup-independent methods (Fig. 2, Fig. S4) and the outgroup-dependent method (Fig. S2). Collectively, our new strains appreciably increase the existing phylogenetic diversity of the PB supergroup. Genome content analysis shows that except for *Bradyrhizobium* sp. ORS285, which is known to use both a *nod*-dependent and -independent strategy for nodulation [9], all PB supergroup members lack the *nod* genes. Our *rpoB* amplicon analysis showed that PB members in all analyzed ecosystems are dominated by the PB Clade 3, whereas the newly isolated Clade 1 showed a low relative abundance but wide habitat distribution (Fig. 1B, Dataset S2). It is worth mentioning that the ASVs assigned to Clade 1 are closely related to the cultured members of Clade 1, but none of them have identical sequences to the cultures (Fig. S5). This suggests that there remains a lot of room to expand the fine-scale diversity within Clade 1.

These three major clades may have diverged functionally as each has a unique set of ecologically relevant genes. For example, genes encoding C4-dicarboxylate transporter (*dctBD*) are universally and exclusively found in Clade 3 (Fig. S6, Dataset S5), potentially enabling Clade 3 strains to acquire C4-dicarboxylate compounds (e.g. malate and succinate) from the host plants to fuel the energy-intensive nitrogen fixation [26]. Other Clade 3-associated genes, though not necessarily exclusively found in Clade 3, include those involved in carbon metabolism and energy conservation processes, such as those encoding histidine utilization enzymes (*hutCFHIU*), glutaconate CoA-transferase (*gctAB*), raffinose/stachyose/melibiose transport system (*msmEFG*), O_2_-independent ubiquinone biosynthesis genes (*ubiDTUV*), and nitrate reductases (*narGHIJ* and *napABCDE*) (Fig. S6, Dataset S5). Specifically, *hut* genes allow using histidine as a source of carbon, nitrogen, and energy for growth and to facilitate nitrogen fixation [27]. The two non-homologous dissimilatory nitrate reductases (*narGHIJ* and *napABCDE*) and the O_2_-independent ubiquinone biosynthesis genes (*ubiDTUV*) are both necessary for denitrification process [28, 29]. The *narGHIJ* carry out nitrate respiration primarily under anaerobic conditions [28], whereas the *napABCDE* reduce nitrate under both anaerobic and aerobic condition [28].

The genes prevalent to Clade 1 include the *mxa* gene cluster (*mxaACDGJKL*), *mgsABC*, and *mdh12* (Fig. S6, Datasets S5 and S6). These genes potentially facilitate the utilization of methanol, which is a waste product during plant-cell wall degradation [30], although a physiological assay did not support this hypothesis (Fig. S7). This suggests that expression of these genes might be regulated by unknown mechanisms. Another unique gene cluster in Clade 1 codes for a protein complex (*exoALOUWY*) (Fig. S6, Datasets S5 and S6) probably involved in the synthesis of the major components of exopolysaccharide (EPS), which may induce the immune response of plants [31, 32]. Plant LysM kinase receptors perceive EPS, and depending on the composition, EPS could either impair or promote symbiosis between rhizobia and plants [31]. Other Clade 1-associated genes include malonate decarboxylase (*mdcABCDEG*), UDP-glucose/iron transport system (*STAR12*), and tungstate transport system (*tupABC*) (Fig. S6, Dataset S5). The presence of these genes implies potential unique carbon metabolism [33] and transporters [34] in this clade.

Clade 2 consists of only two strains, making our findings on their gene composition, inconclusive. However, compared to Clade 1 and 3, these two strains in Clade 2 uniquely have a gene cluster for the type VI secretion system (T6SS) (Fig. S6, Dataset S5), which is known to influence bacterial competitiveness and symbiosis with eukaryotes [35, 36]. The effects on symbiosis with plants may be closely related to the effectors secreted by T6SS that interact with the host plant and surrounding microbiota [35]. Other specific genes in these two strains include pyruvate ferredoxin oxidoreductase (*porABC*) (Fig. S6, Dataset S5), which exclusively supports fermentation under anaerobic conditions and releases energy at the same time [37].

### Fine-scale population structure of the Photosynthetic supergroup of *Bradyrhizobium*

Apart from the broad diversity of the PB supergroup, we further asked whether fine-scale phylogenetic differentiation correlates with the habitats where the PB supergroup members were found. Specifically, bacterial isolation was performed from endosphere, rhizosphere, and bulk soil for each plant, collectively giving rise to 156, 54, and 53 PB strains, respectively (Dataset S3). To facilitate the correlative analysis between fine-scale phylogenetic groups and the habitats, we assigned isolates into populations defined by PopCOGenT (reported as “main cluster” or MC) [24]. As bacterial members within a main cluster have significantly higher recombination rate than those across main clusters, “population” defined here aligns with the “species” in higher eukaryotes and differs from the concept in microbial ecology where a population typically refers to a collection of closely related strains residing in the same ecological habitat. Additionally, PopCOGenT can detect subpopulations (reported as “subclusters”), which are under ongoing differentiation within a population. To avoid confusion, we use main cluster and subcluster throughout.

Using PopCOGenT, we show that (i) all strains in Clade 1 share the membership of a single main cluster (MC1), (ii) the two strains in Clade 2 each form a distinct main cluster (MC id not given), though they may be deemed as members of a single operational species as their genome-wide average nucleotide identity (ANI) is 96.5% (Fig. S8, Dataset S7), exceeding the species threshold of 95%, and (iii) strains from Clade 3 fall into numerous main clusters, among which, MC2, MC3, MC4, MC5, MC6, MC8, MC9, and MC11 are each exclusively comprised of the new strains, whereas MC7, MC10, and the remaining six unassigned isolates each forming a distinct main cluster are publicly available (Fig. 2). In Clade 3, the within- and between-MC similarity is above and below 95% ANI (Fig. S8, Dataset S7), respectively, consistent with the operational species threshold of 95% ANI [38], whereas the 16S rRNA gene shows little divergence with the between-MC similarity generally above 99% (Fig. S8, Dataset S8). Based on the available samples, intra-MC subdivision occurred within MC1, MC2, MC3, and MC5. We found that each main cluster and subcluster have members sampled from different plants and habitats (Fig. 2), suggesting that the PB main clusters sampled from the rice field are not genetically subdivided according to the physical separation between the plant individuals or following the habitat separation between endosphere, rhizosphere, and bulk soil.

We found associations between important metabolic pathways and MC identity. Although the PB supergroup was named by the presence of the photosynthetic genes [3], these genes are exclusively and universally found in MC2, MC5, MC6, MC7, MC8, MC9, MC10, MC11, and the unassigned individuals in Clade 3 but completely missing from MC3 and MC4 of Clade 3, Clade 2, and Clade 1 (MC1) (Fig. 2, Dataset S6). Our result indicates that photosynthesis is not a characteristic trait that defines the PB supergroup. Also interesting is the prevalence of a unique Type III secretion system (T3SS) in MC2, MC3, and MC4 but completely missing in other main clusters (Fig. 2, Fig. S9B and C, Dataset S6). It is one of the six T3SS subtypes identified from known *Bradyrhizobium* phylotypes [39]. It was previously identified only in the PB strain *Bradyrhizobium oligotrophicum* S58 (thus named “S58-T3SS” subtype) and its function remains unknown [39]. Because the general function of T3SS is to translocate effector proteins into host cells that modulate the host immune response [40], it cannot be excluded that this T3SS type specifically identified in PB members plays an important role during their interaction with their host plants (*Aeschynomene* spp. and rice).

### Nodulation ability in *Aeschynomene indica* is a conserved trait shared by Photosynthetic supergroup of *Bradyrhizobium* but differs between the major clades

All PB supergroup members have a *nif* gene cluster necessary for nitrogen fixation. Duplication of the *nifH* gene which encodes one of the structural proteins of the nitrogenase enzyme complex was observed in all PB strains (Fig. S9A), but the functional consequence is unknown. Across the PB supergroup members, an important difference was observed regarding how the *nif* genes are structured. In most main clusters, all *nif* genes are co-located, along with other genes (e.g. *sufBCDX*, *glbO*, and *fixABCX*) potentially involved in nitrogen fixation. This is not the case for a few members such as some basal lineages (e.g. *Bradyrhizobium* sp. LMG 8443) and some members assigned to several main clusters (e.g. SZCCHNR1015 in MC5, *Bradyrhizobium* sp. 83 002 in MC7, and *Bradyrhizobium* sp. LMG 8443 in MC10), where the *nif* genes are present in two contigs. This is likely due to structural rearrangement, but a DNA assembly artifact cannot be ruled out.

A common feature of the PB *nif* cluster is the universal presence of the *nifV* gene encoding for a homocitrate synthase that is involved in the biosynthesis of the nitrogenase cofactor (FeMo-Co), which is absent in most other rhizobial lineages [41, 42]. An acetylene reduction assay was performed on 28 representative PB strains, which cover all three major clades, the newly identified main clusters (MC1 to MC6), most of the identified subclusters, and the model PB strain ORS278 as a control. The result confirmed the ability of the PB strains to fix nitrogen under free-living conditions (Fig. S10A), a trait that is absent in most *nod*-carrying rhizobia [41]*.*

**Figure 3:**
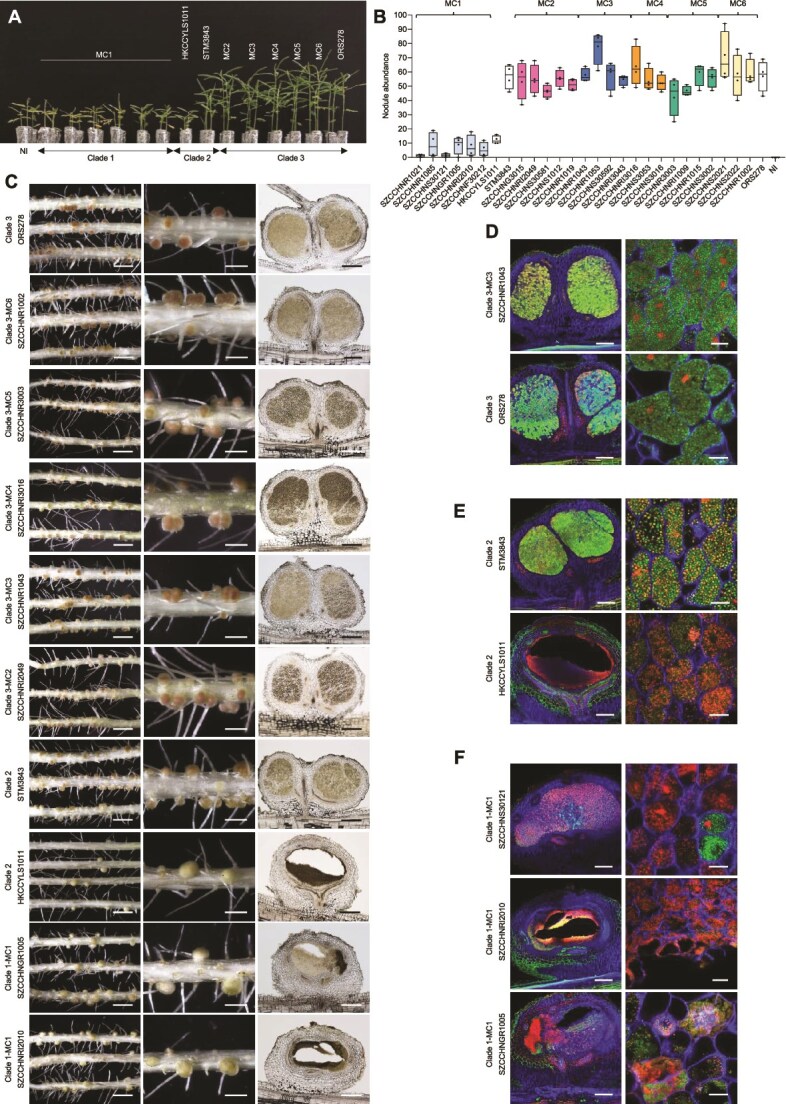
Symbiotic properties of representative strains covering the broad and fine-scale diversity of the PB supergroup. (A) Comparison of growth of the *A. indica* plants (leaf phenotype) non-inoculated (NI) or inoculated with different representative strains of PB. The six MC1 representative strains are presented in the following order: SZCCHNR1021, SZCCHNR1085, SZCCHNS30121, SZCCHNGR1005, SZCCHNRI2010, and SZCCHNF30212. For the other MCs, only one representative strain is shown: SZCCHNRI2049 (MC2), SZCCHNS30592 (MC3), SZCCHNRI3016 (MC4), SZCCHNS3002 (MC5), and SZCCHNS2021 (MC6). Photos were taken 17 days after inoculation (dpi). (B) Nodule abundance on *A. indica* plants induced by different representative strains of PB at 17 dpi. Box plots show the results of one of the two experiments performed independently (four plants each, eight in total). (C) Symbiotic phenotypes of some representative strains tested on *A. indica*. Column 1 and 2: photo of roots and nodules. Scale bars: column 1, 0.5 cm; column 2, 0.2 cm. Column 3: micro-sections of nodules observed using light microscopy. Scale bars: 250 μm. (D) Confocal microscopy images of micro-sections of nodules elicited by Clade 3 strains. The nodules formed by SZCCHNR1043 from MC3 and ORS278 are shown as examples. The central nodule tissue is intracellularly infected with live spherical bacteroids stained green by SYTO9. (E) Confocal microscopy images of micro-section of nodules elicited by Clade 2 strains (STM3843 and HKCCYLS1011). The nodules elicited by STM3843 look normal, but a mix of live and dead bacteria (stained red by propidium iodide) can be noticed whereas the nodules formed by HKCCYLS1011 displayed a central infected tissue that is digested. (F) Confocal microscopy images of micro-sectioned nodules elicited by Clade 1 strains displaying different phenotypes: Nodule containing mainly dead bacteria (SZCCHNS30121), nodule with a completely digested central tissue (SZCCHNRI2010); and nodule with a necrotic area and a digested zone (SZCCHNGR1005). Scale bars for (D), (E), and (F): column 1, 200 μm; column 2, 10 μm.

The plant inoculation experiments showed that all 28 representative strains were able to induce nodules on *A. indica* (Fig. 3B and C). However, a difference in symbiotic efficiency was observed between the major clades in terms of growth development, nodule number, and nitrogen fixation. Clade 3 strains had the same symbiotic properties as the model strain ORS278, inducing many nitrogen-fixing nodules that stimulated the growth of the plants (Fig. 3A, B, and D, Figs S10B and S11). In contrast, Clade 1 strains formed considerably fewer nodules compared to Clade 3 strains and the plant nitrogenase activity was lower (Fig. 3B, Fig. S10B). Microscopic analysis showed that the nodules elicited by Clade 1 strains displayed multiple aberrant phenotypes: (i) some nodules contained necrotic areas, (ii) in others the central tissue was completely digested, and finally, (iii) the nodules that displayed less drastic symptoms contained mainly dead bacteria (Fig. 3C and F). These observations indicate that besides inducing fewer nodules, Clade 1 strains cannot maintain an effective chronic infection, which explains why they have no beneficial effect on plant growth (Fig. 3A, C, and F, Fig. S11A). Clade 2 represented by only two strains STM3843 and HKCCYLS1011 exhibits a transitional pattern of phenotype. The number (Fig. 3B) and phenotype (Fig. 3C and E) of the nodules elicited by strain STM3843 are comparable to those of Clade 3 strains (Fig. 3B, C, and D) though stimulation of plant growth is less (Fig. 3A), whereas the nodules stimulated by strain HKCCYLS1011 (Fig. 3B, C, and E) are like those of Clade 1 strains (Fig. 3B, C, and F) and no effect on plant growth was observed (Fig. 3A).

The symbiotic efficiency differences observed between the major clades may result from an inability of the Clade 1 strains to cope with the plant’s immune response, and/or an over-induction of the plant’s defense mechanisms due to the absence or not properly recognized symbiotic signal(s). For example, Clade 1 strains possess *exoALOUWY* for the synthesis of exopolysaccharides that could induce plant immune responses (Fig. S6). In contrast, most Clade 3 strains possess photosynthetic genes and T3SS (Fig. 2), which could promote PB-*Aeschynomene* symbiosis. Besides, they contain many genes involved in carbon transport, metabolism, and energy production, such as *dctBD*, *hutCFHIU*, providing energy for nitrogen fixation and promoting plant growth (Fig. S6). Despite our poor understanding of those three clades, it is remarkable that all strains from the PB supergroup, which come from different origins and geographical locations, are capable of nodulating *A. indica*. Thus, the ability to develop an NF-independent nodulation with *Aeschynomene* spp. appears to be a common trait of PB members. Thus, unlike the *nod* genes of other rhizobial lineages which are accessory genes acquired by horizontal transfer, the genes governing NF-independent symbiosis may be essential genes belonging to the core genome of the PB supergroup.

## Concluding remarks

The results of *rpoB* amplicon sequencing of samples from forest, grassland, rice, and maize field indicate that members of the PB supergroup are enriched in rice field. By large-scale isolation and genome sequencing, we report a new, deep-branching clade, thereby greatly expanding the phylogenetic diversity of the cultured members of PB. An important finding is that although all assayed phylogenetically diverse PB strains can nodulate *Aeschynomene* spp., the phylogenetic divergence of the three major clades correlates nicely with their symbiotic efficiency. It is therefore likely that the ability to establish symbiosis with *Aeschynomene* plants, which often grow in the same wetlands as rice, is a key factor that shapes the broad diversity of the PB supergroup. At the main cluster level, no significant difference in symbiotic efficiency was observed within Clade 3, suggesting that other traits such as photosynthesis and T3SS that are uniquely associated with some but not all main clusters, might be among the important drivers of MC-level genetic differentiation and ecological adaptation. Collectively, our study provides insights into the ecology and evolution of the Photosynthetic supergroup within the globally dominant soil bacteria *Bradyrhizobium*, and additionally serves as a prime example that links deep phylogenetic diversity of an ecologically relevant bacterial group to their major phenotypic and functional diversity.

## Supplementary Material

SI_Figures_V2_wraf030

SI_wraf030

Supplymentary_dataset_wraf030

## Data Availability

The genomic sequences and raw reads of the 263 newly sequenced *Bradyrhizobium* isolates have been uploaded to NCBI (Project ID: PRJNA983111).
