# Supplementary material for: Correlating phylogenetic and functional diversity of the nod-free but nodulating Bradyrhizobium phylogroup
Source: ISME J. 2025 Feb 17;19(1):wraf030. doi: 10.1093/ismejo/wraf030 (PMC11973431; doi:10.1093/ismejo/wraf030)
Supplement: SI_Figures_V2_wraf030 [file si_figures_v2_wraf030.pdf]

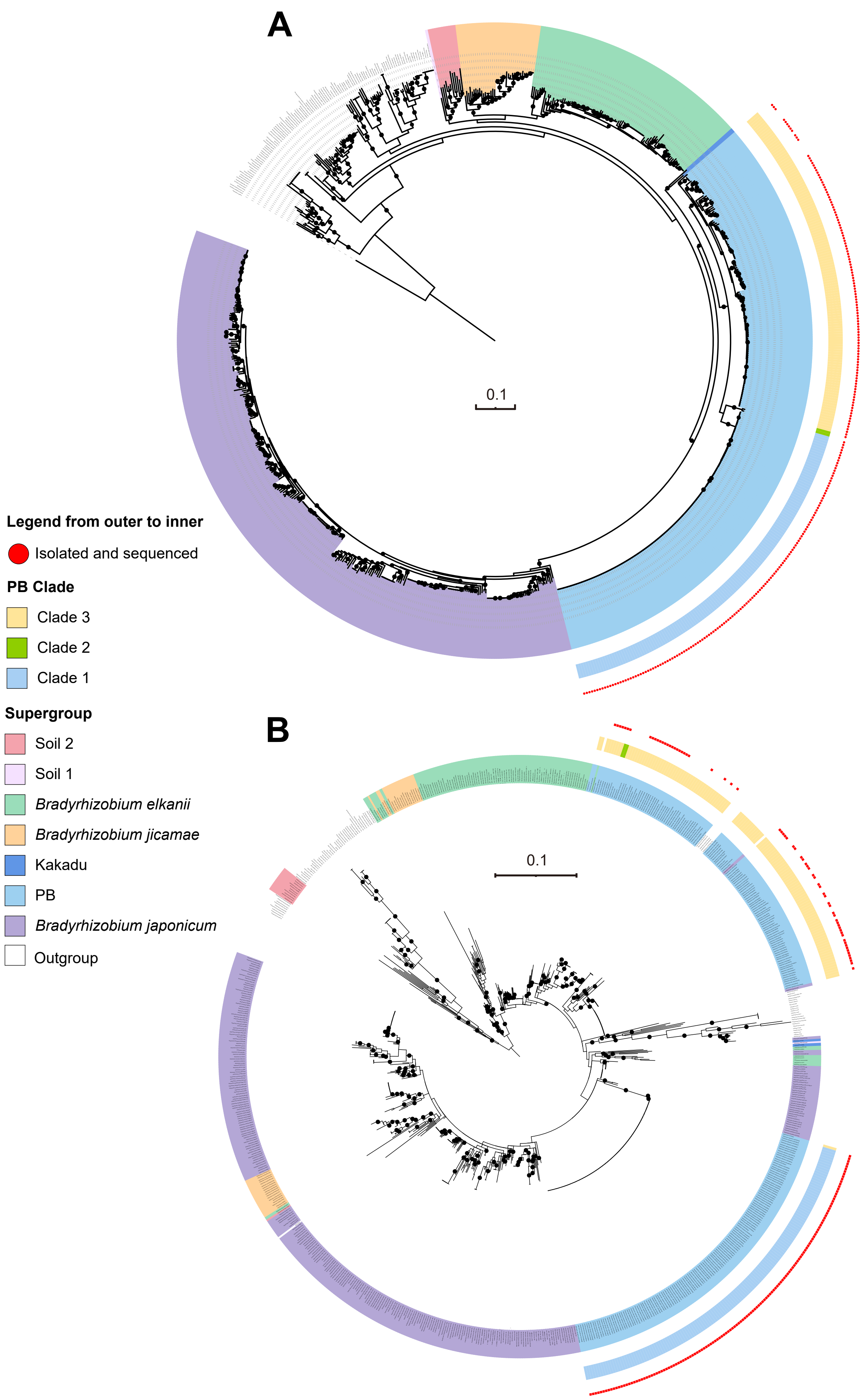

Figure S1. *RpoB* gene tree constructed by full length (A) and amplified region (B). Strains from Xanthobacteraceae were used as an outgroup. The black circles on the nodes indicate ultrafast bootstrap values higher than or equal to 95% calculated by IQ-Tree. The 263 PB strains sequenced in the present study are indicated by red dots in the outermost layer surrounding the tree. The scale bar inside each tree represents the number of substitutions per site.

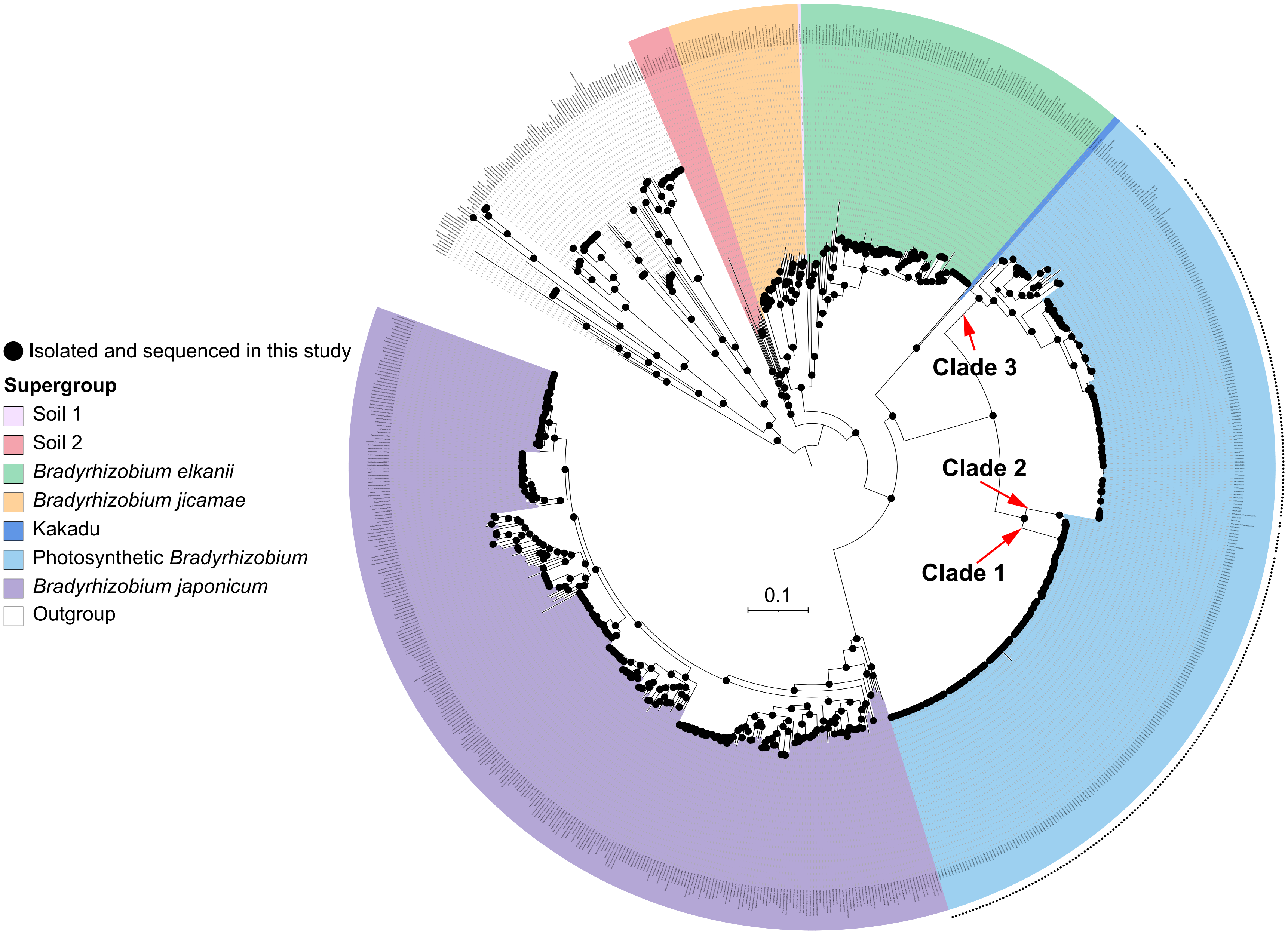

Figure S2. The maximum-likelihood phylogenomic tree of *Bradyrhizobium*. Strains from Xanthobacteraceae were used as an outgroup. The tree was constructed using the 123 orthologous genes identified in a previous study (Tao et al., 2021). The black circles on the nodes indicate ultrafast bootstrap values higher than or equal to 95% calculated by IQ-Tree. The 263 PB strains sequenced in the present study are indicated by black dots in the outermost layer surrounding the tree. The scale bar indicates the number of substitutions per site.

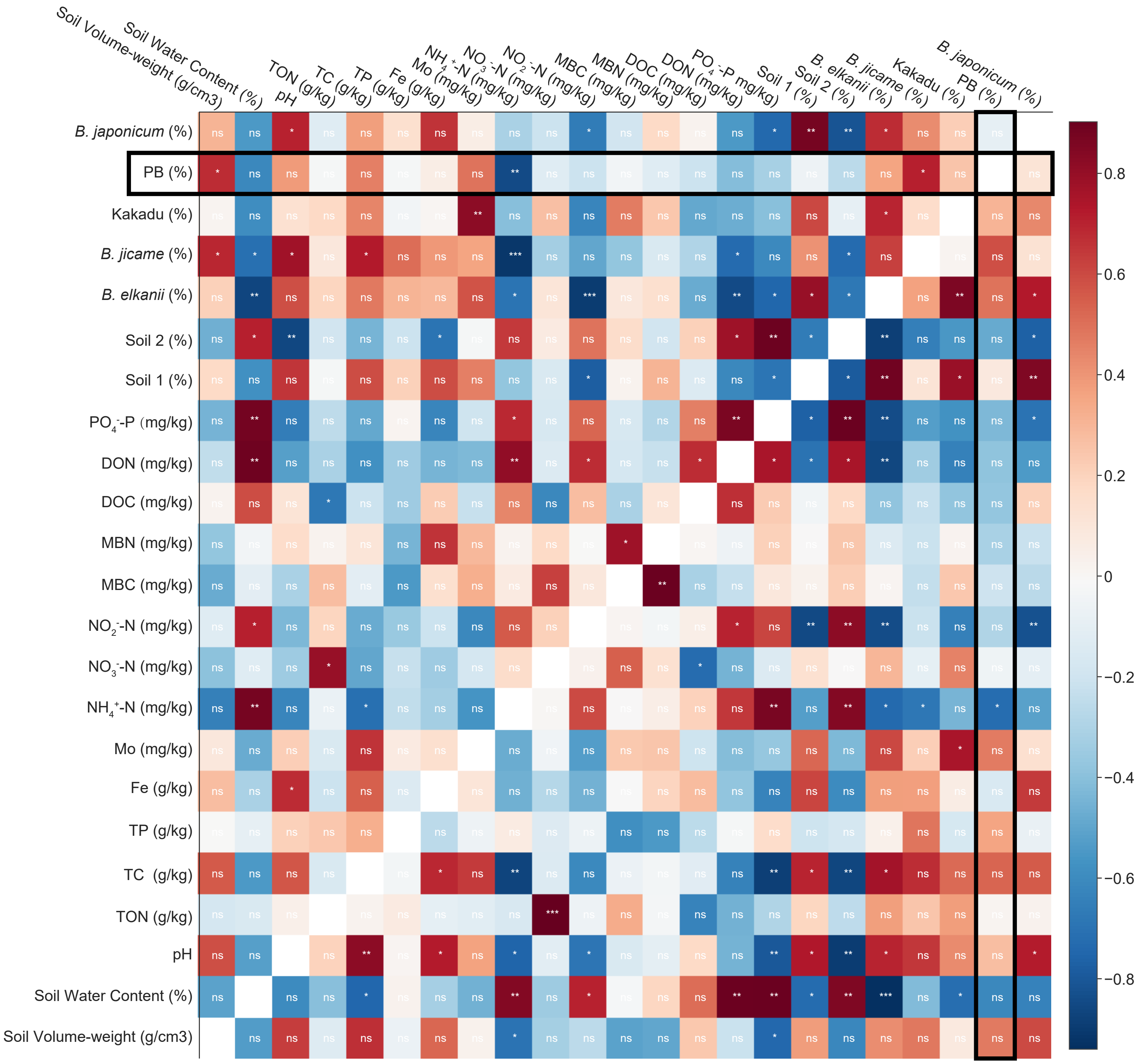

Figure S3. Correlation analysis between *Bradyrhizobium* supergroups and basic soil characteristics. The upper left and lower right triangles are Spearman and Pearson correlation analysis, respectively. Correlation coefficient is shown (ns represents non-significant difference). Blue-tinted squares represent negative correlations, while red-tinted squares represent positive correlations. The boldness of the colour represents the strength of the relationship between the variables, with stronger correlations having bolder colours. The number of asterisks denote the significance of the correlation: * refers to $P < 0.05$, ** refers to $P < 0.01$, *** refers to $P < 0.001$.

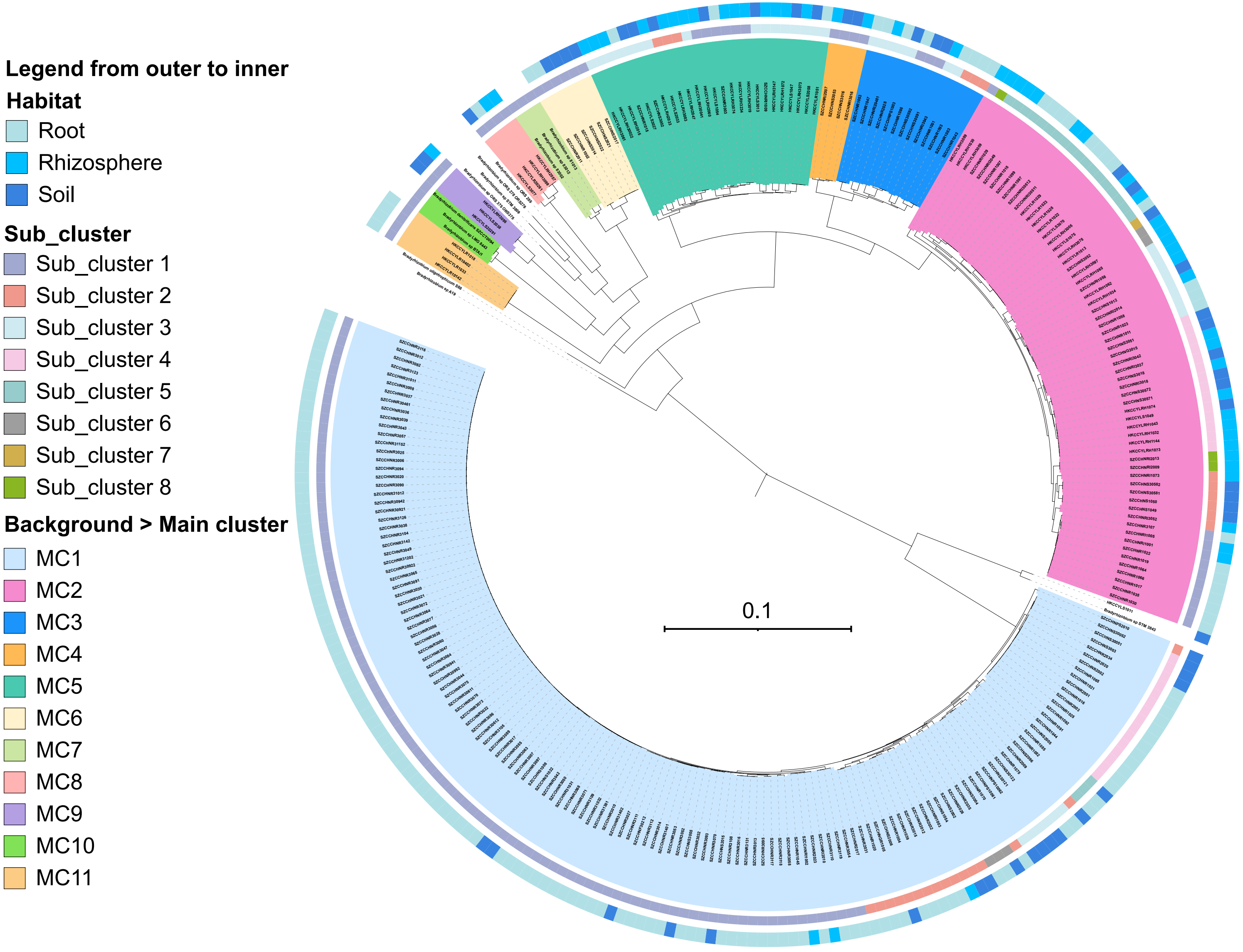

Figure S4. The phylogenomic tree of the Photosynthetic *Bradyrhizobium* is based on the minimal ancestor deviation (MAD) rooting method. Solid circles in the phylogeny indicate nodes with IQ-Tree's ultrafast bootstrap values ≥ 95%. The scale bar indicates the number of substitutions per site.

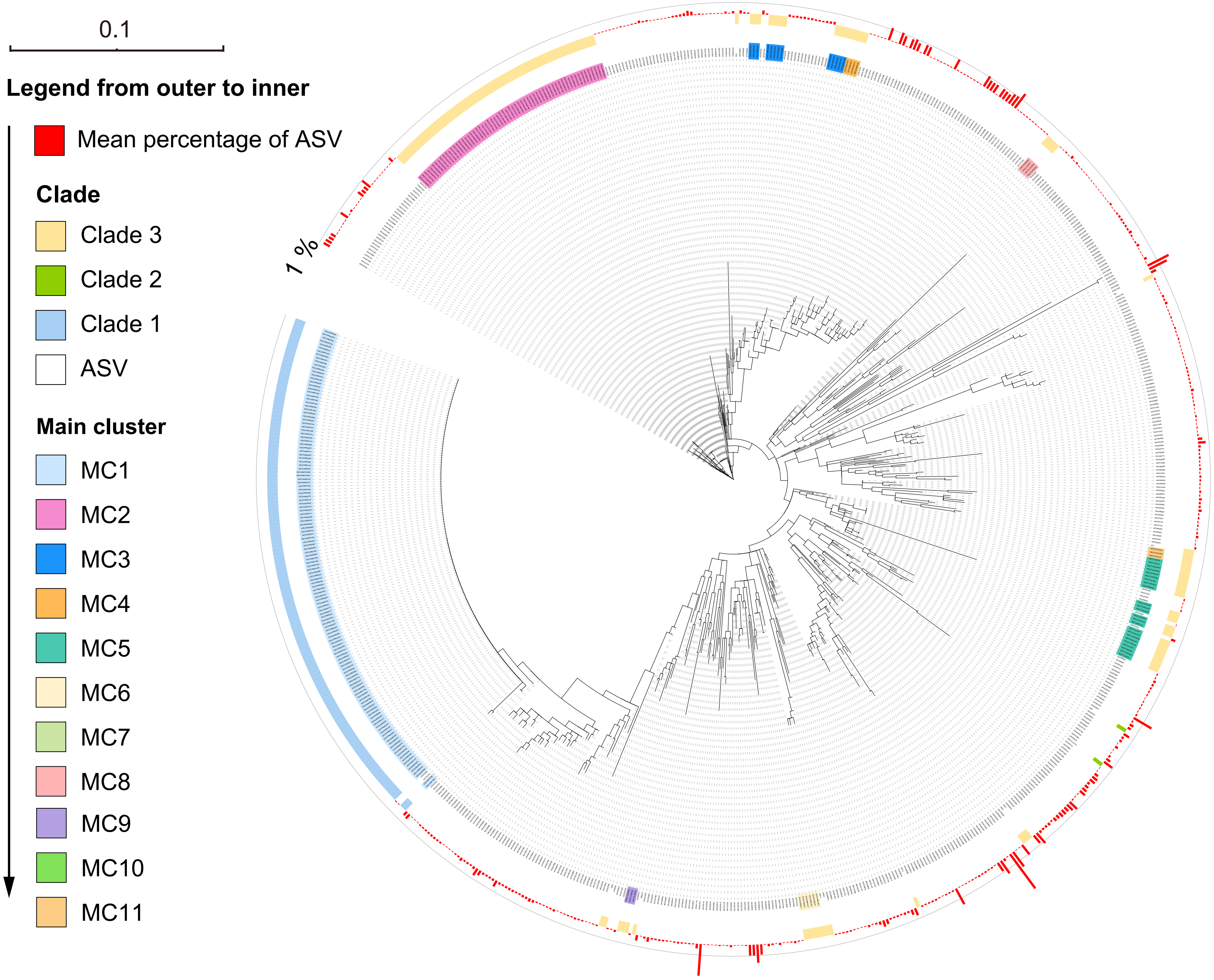

Figure S5. The ASVs (amplicon sequence variants) and *rpoB* genes (amplified regions from Photosynthetic *Bradyrhizobium* genomes) tree. This gene tree was rooted by the minimum variance (MV) method. The 276 *rpoB* genes from PB genomes in this study were divided into each clade and main cluster (MC) according to Fig. 2. The scale bar indicates the number of substitutions per site.

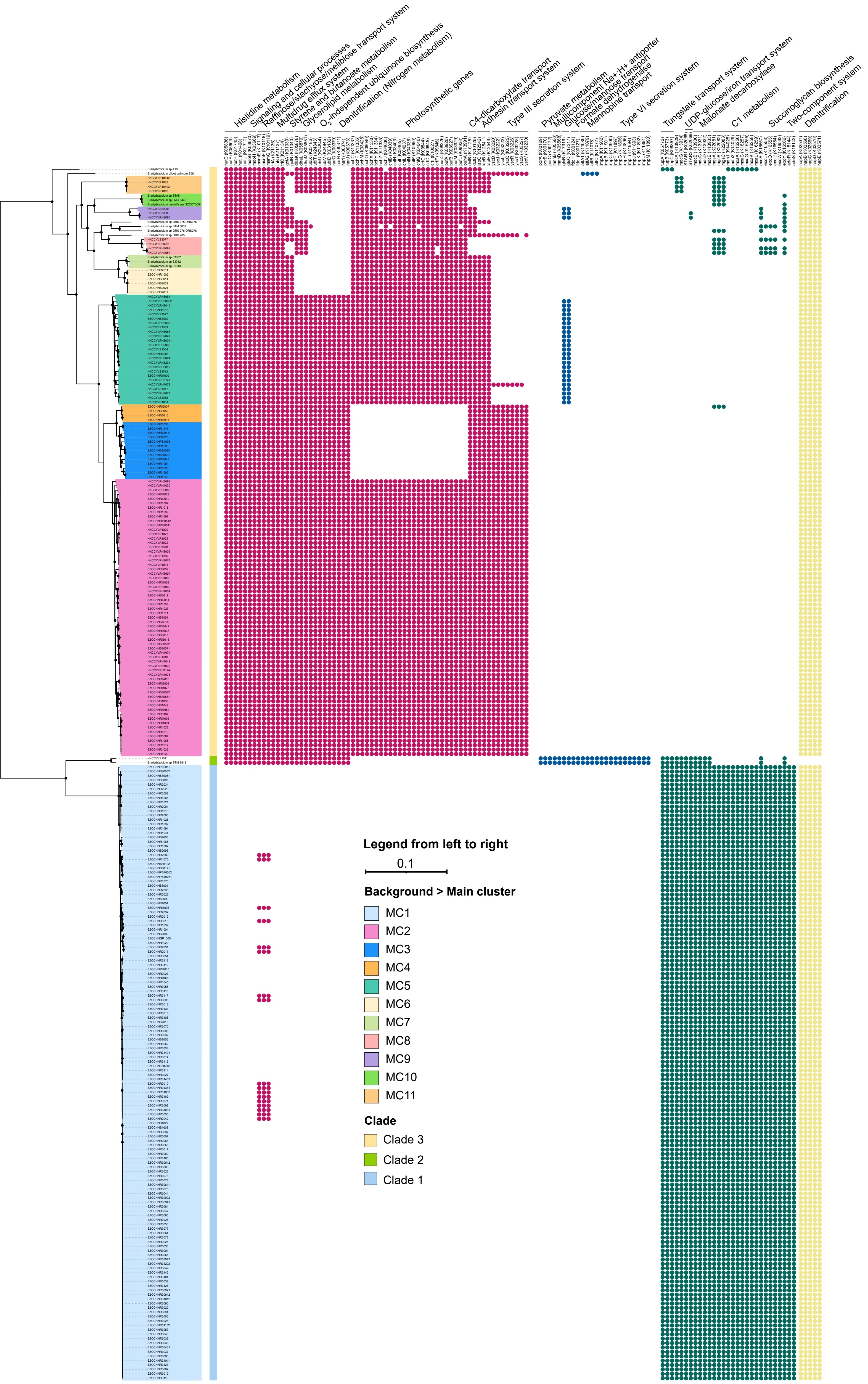

Figure S6. The phyletic pattern of specific genes between three PB clades. The solid circles and blank in the right panel represent the presence and absence of the genes, respectively. The genome tree in the left panel was displayed in rectangular mode. Detailed information of specific genes was shown in Dataset S5. Histidine metabolism *hutCFHIU*, signaling and cellular processes *mdoGH*, raffinose/stachyose/melibiose transport system *msmEFG*, multidrug efflux system *triABC*, styrene and butanoate metabolism *gctAB*, glycerolipid metabolism *dhaKLM*, O_2-independent ubiquinone biosynthesis *ubiXTUVD*, nitrogen metabolism (denitrification) *narGHIJ napABCDE*, photosynthetic genes 1) porphyrin metabolism *bchCFMOXYZJ* and *chlBHILNPG* 2) carotenoid biosynthesis *crtCDIF* 3) light-harvesting complex *pufAB* 4) photosynthetic reaction center *pufML* and *puhA*, C4-dicarboxylate transport *dctBD*, adhesin transport system *lapBCE*, Type III secretion system, pyruvate metabolism *porABC*, multicomponent Na^+:H^+ antiporter *mnhBD*, glucose/mannose transport system *gtsBC*, formate dehydrogenase *fdoHI*, mannopine transport system *attA1A2BC*, Type VI secretion system, tungstate transport system *tupABC*, UDP-glucose/iron transport system *fetAB*, malonate decarboxylase *mdcABCDEG*, C1 metabolism *mgsABC mxaFI* and *mxaACGKL*, succinoglycan biosynthesis *exoALOUWY*, two-component system *adeRS*. The scale bar represents the number of subsubstitutions per site.

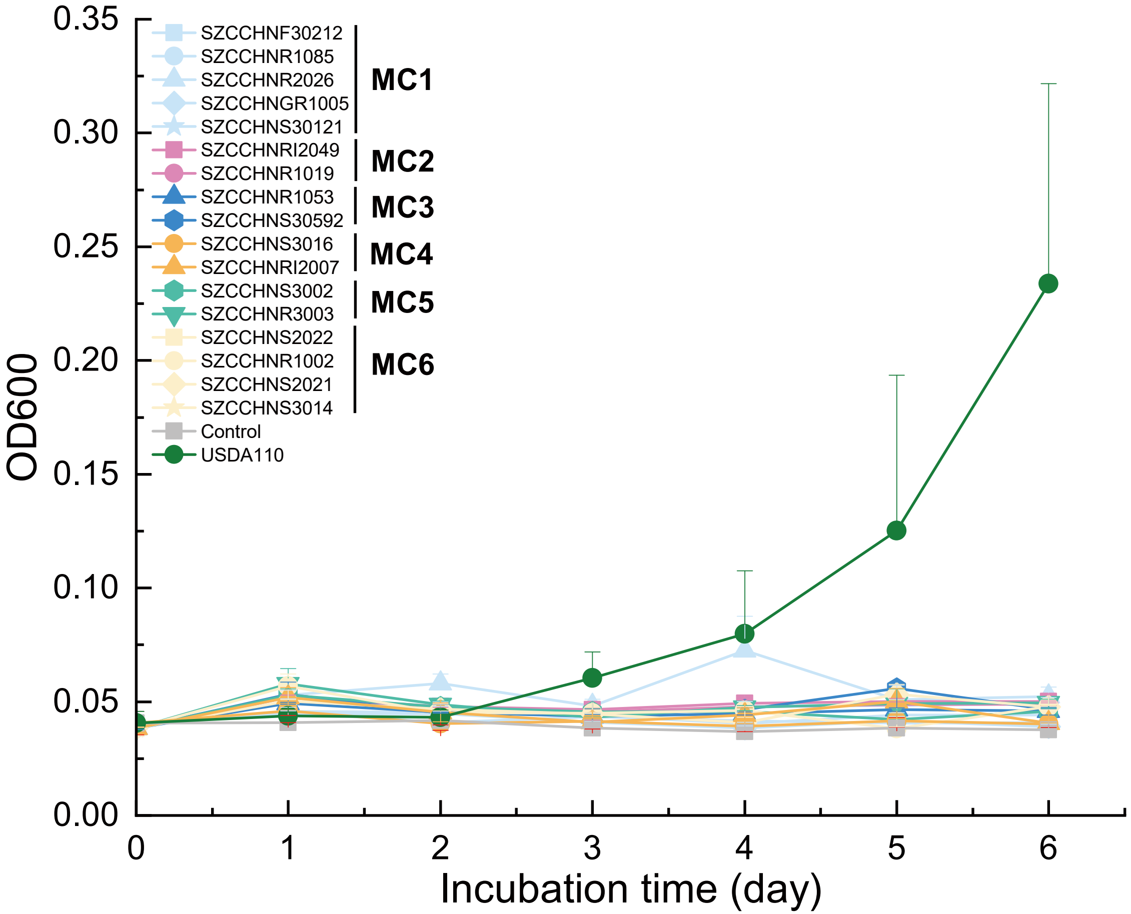

Figure S7. Growth of representative strains from the populations delineated by PopCOGenT for the PB of *Bradyrhizobium* on methanol as a sole carbon source, with the presence of lanthanide (Ln) species (Ce^{2+}, 30 uM). The reference strain *Bradyrhizobium diazoefficiens* USDA110 was used as a positive control. Error bars indicate the standard deviation of the mean from three replicates.

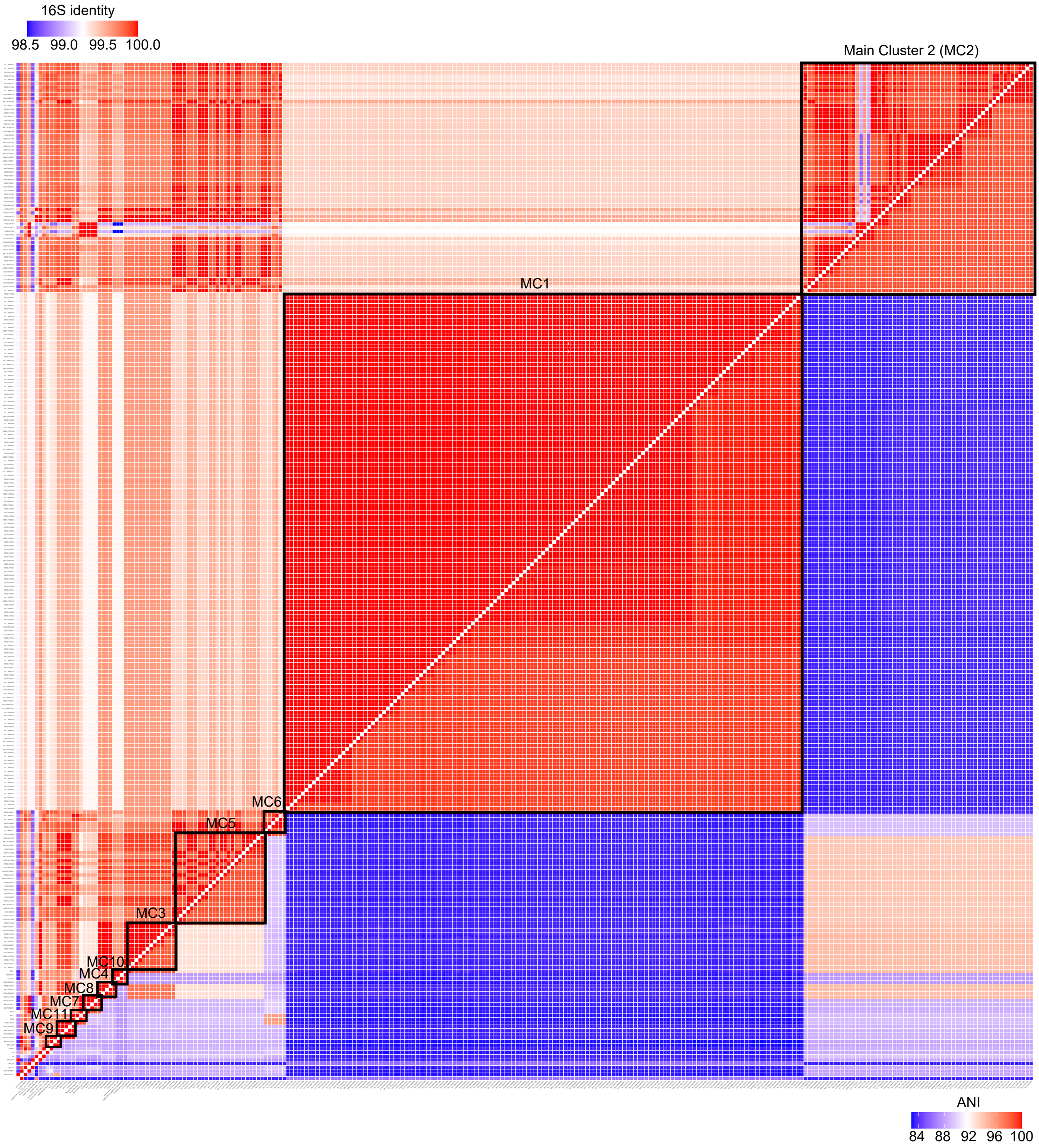

Figure S8. The heatmap of the pairwise identity of 16S rRNA genes and the whole-genome average nucleotide identity (ANI) of all PB members.

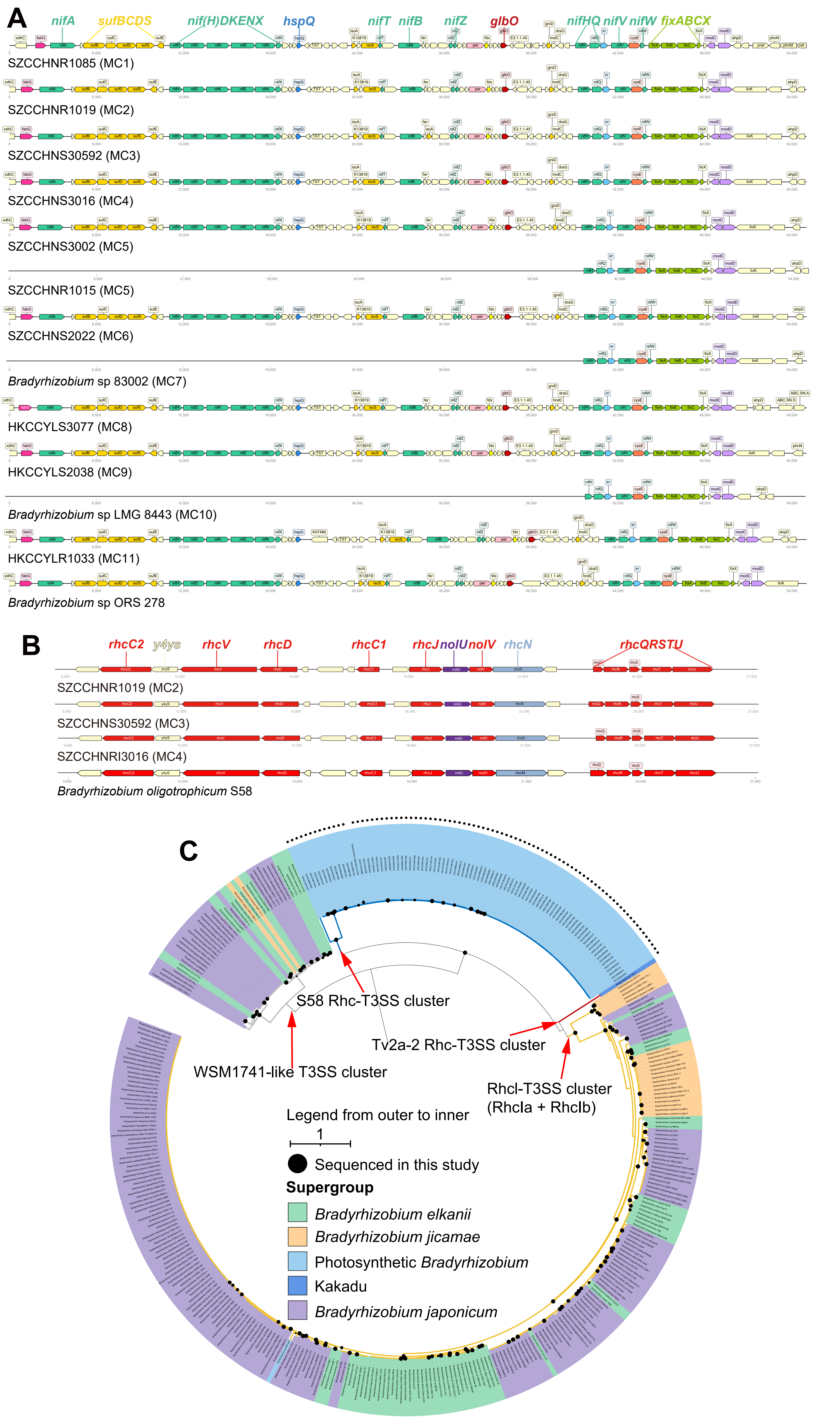

Figure S9. Comparison of the genomic context of the *nif* gene cluster (*nif* island) (A) and T3SS gene cluster (B) in the representative strains of the Photosynthetic *Bradyrhizobium*. Gene functions are distinguished by different colors. The visualization of gene arrangement is performed with DNA-features-viewer v3.0.3 (Zulkower and Rosser, 2020). (C) The phylogenetic tree of the *rhcN* protein from *Bradyrhizobium*. The *rhcN* families were defined according to Teulet et al. (2020). The gene tree was rooted using the minimum variance (MV) method. The different colored branches correspond to the distinct genetic organization of the T3SS clusters to which the *rhcN* gene belongs. The *rhcN* in the strain *Bradyrhziobium* sp. 36 is not shown as it belongs to a different type of T3SS, likely a result of HGT from distantly related bacteria according to Teulet et al. (2020). Black circles in the phylogeny indicate nodes with IQ-Tree's ultrafast bootstrap values ≥95%. The scale bar indicates the number of substitutions per site.

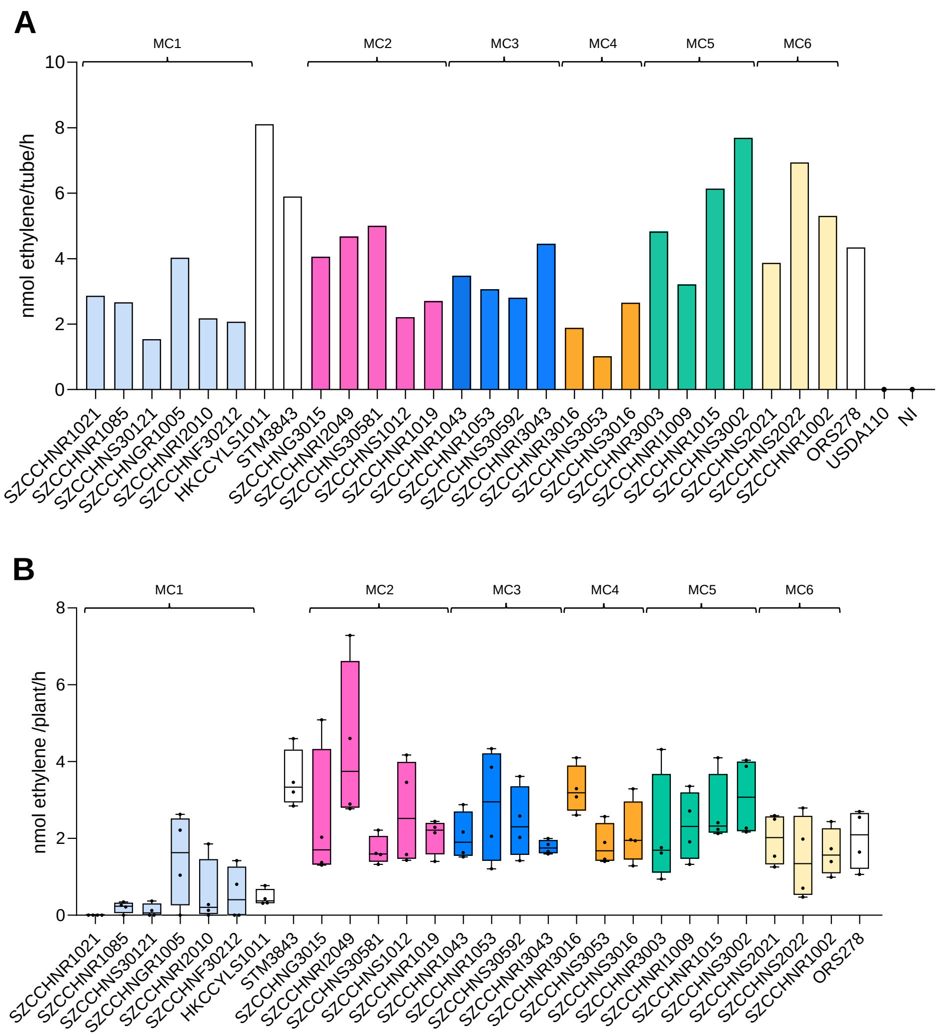

Figure S10. Ability of several representative strains of the main clusters identified in PB supergroup to fix nitrogen during their free-living and symbiotic states. (A) Free-living nitrogen fixation after 8 days of culture in vacutainer tube. Two replicates of each strain were performed under this condition and their average values were used for this figure. (B) Nitrogen fixation of *A. indica* plants inoculated with different PB representative strains at 17 days post-inoculation. In (A) and (B), ORS278 is used as a positive control and USDA110 (a member of *B. japonicum* supergroup) is used as a negative control in (A). NI: non inoculated. MC: Main Cluster

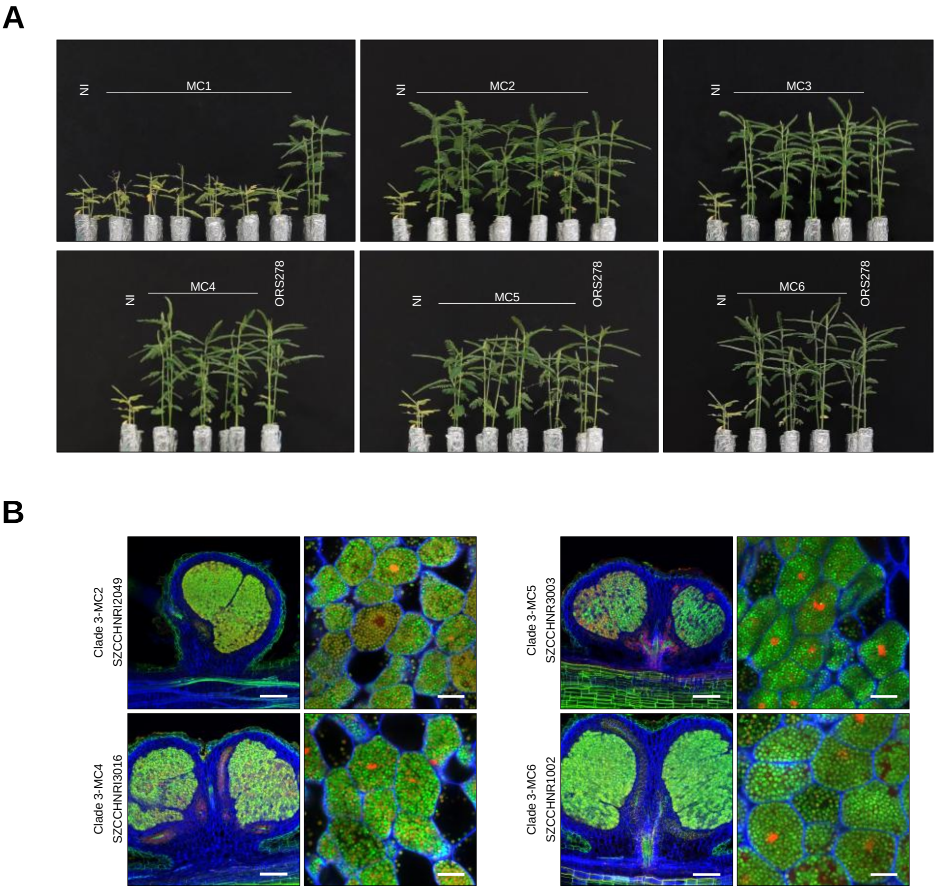

Figure S11. Complementary data of Fig. 3 showing symbiotic properties of several representative strains of the main clusters identified in PB supergroup. (A) Comparison of the growth of the *A. india* plant (leaf phenotype) non-inoculated (NI) or inoculated with different representative strains of PB. All representative strains tested from each MC are present in this order : MC1 - SZCCHNR1021; SZCCHNR1085; SZCCHNS30121; SZCCHNGR1005; SZCCHNRI2010; SZCCHNF30212; MC2 - SZCCHNG3015; SZCCHNRI2049; SZCCHNS30581; SZCCHNS1012; SZCCHNR1019; MC3 - SZCCHNR1043; SZCCHNR1053; SZCCHNS30592; SZCCHNRI3043; MC4 - SZCCHNRI3016; SZCCHNS3053; SZCCHNS3016; MC5 - SZCCHNR3003; SZCCHNRI1009; SZCCHNR1015; SZCCHNS3002 and MC6 - SZCCHNS2021; SZCCHNS2022; SZCCHNR1002. ORS278 is used as control. (B) Confocal microscopy images of micro-section of nodules elicited by the other Clade 3 strains tested after staining with SYTO9 (green, live bacteria), propidium iodide (red, infected plant nuclei and dead bacteria or bacteria with compromised membranes) and calcofluor (blue, plant cell wall). Scale bars: column 1, 200 µm; column 2, 10 µm.

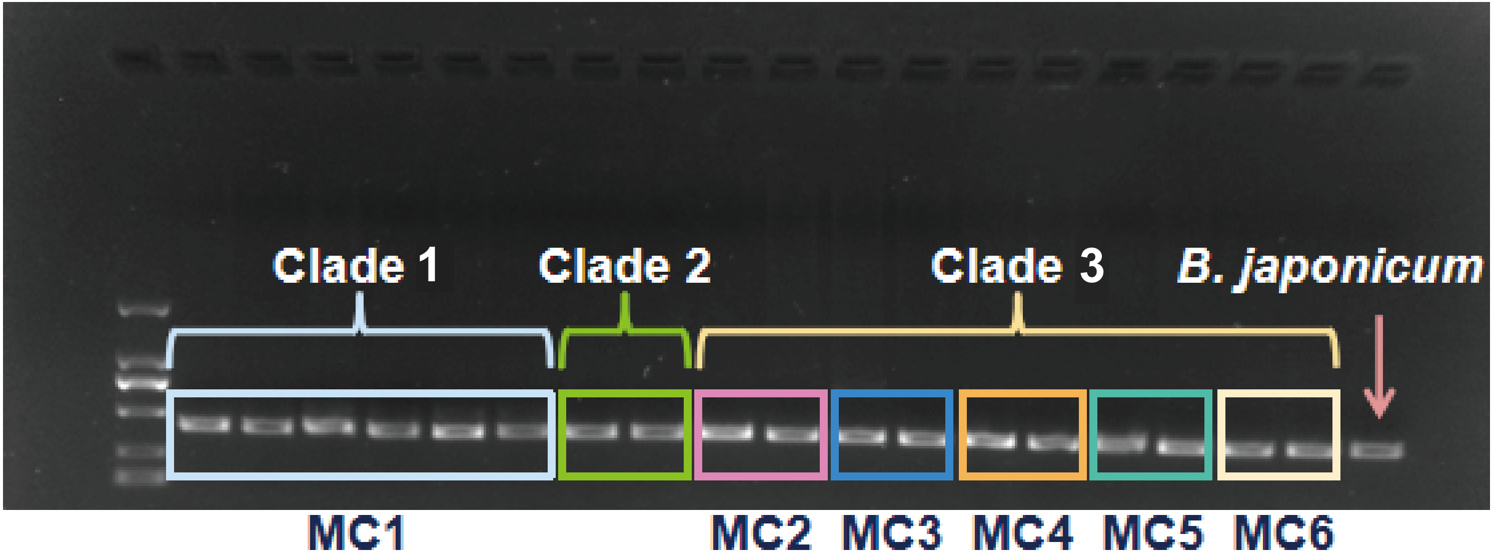

Figure S12. Gel electrophoresis image of DNA from PB strains amplified with the specific *rpoB* primer set BR2106F/BR2516R.
