# Supplementary material for: Correlating phylogenetic and functional diversity of the nod-free but nodulating Bradyrhizobium phylogroup
Source: ISME J. 2025 Feb 17;19(1):wraf030. doi: 10.1093/ismejo/wraf030 (PMC11973431; doi:10.1093/ismejo/wraf030)
Supplement: SI_wraf030 [file si_wraf030.docx]

**Supplementary Information**

**Correlating phylogenetic and functional diversity of the *nod*-free but nodulating *Bradyrhizobium* phylogroup**

Lu Ling^^^, Alicia Camuel^^^, Sishuo Wang, Xiaojun Wang, Tianhua Liao, Jinjin Tao, Xingqin LIN, Nico Nouwen, Eric Giraud^*^, Haiwei Luo^*^

**^^^These are co-first authors**: Lu Ling, Alicia Camuel

^*^**These are co-corresponding authors:** Eric Giraud ([eric.giraud@ird.fr](mailto:eric.giraud@ird.fr)), Haiwei Luo ([haiweiluo@cuhk.edu.hk](mailto:haiweiluo@cuhk.edu.hk))

**This PDF file includes:**

**Supplementary Text.** Methods

**Supplementary references.**

**Figure S1 to S12.**

**Supplementary Text: Methods**

## Sample niche classification

Excess soil was vigorously shaken away from the plant roots to remove loose soil, leaving approximately 1 mm of soil attached to the roots, which constitutes the rhizosphere compartment [1]. To separate the 1 mm of rhizosphere soil directly from the roots, roots were cut into 5 cm lengths and two grams were placed in a sterile flask containing 50 mL of sterile phosphate buffered saline (PBS) solution and vortexed several times (15 seconds). The PBS solution used to wash the roots was transferred to a 50 mL Falcon tube and then centrifuged at 10,000 x g for one minute. The supernatant was discarded, and the remaining soil samples were saved as rhizosphere samples [1]. The roots were then resuspended and vortexed three times (15 seconds each) before being transferred to new Falcon tubes as root samples. Soil away from the root was stored as bulk soil.

## *Bradyrhizobium* isolation

The root, rhizosphere, and bulk soil samples were placed in the incubation chamber for three to four days at 28°C and 70% relative humidity under the day/night (16/8 h) cycle. All root samples were washed with sterile Milli-Q water to remove any adhering soil, then subjected to the surface sterilization by immersion in 75% ethanol for three minutes, followed by 5% sodium hypochlorite for five minutes, and rinsed five times with sterile distilled water [2]. The washed roots were then aseptically dried on the clean bench before being ground in a sterilized mortar and pestle with PBS buffer (pH 6.8). A series of fivefold and tenfold dilutions were prepared and then inoculated (100 µL) on modified arabinose-gluconate (MAG) media without any nitrogen source (1.0 g DL-arabinose, 1.0 g sodium gluconate, 1.0 g yeast extract, 2 mL KH_2_PO_4_ solution (110 g/L), 4 mL Na_2_SO_4_ solution (62.5 g/L), 1 mL MgSO_4_•7H_2_O solution (180 g/L), 1 mL CaCl_2_ solution (13 g/L), 1 mL FeCl_3_•6H_2_O solution (6.7 g/L), and 15 g agar were added. The medium was made up to 1 L with Milli-Q water, the pH was adjusted to 6.6 with KOH, and then autoclaved at 121 ℃ for 15 - 30 minutes.) [3]. To prepare soil inoculations, 5.0 g of fresh soil was mixed with 45 mL of sterile deionized water in a 50 mL Falcon tube. One mL of soil suspension was serially diluted 10-fold after mixing with a vortex mixer, and 100 μL of diluted samples were spread on the MAG media [3]. The isolation plates were then placed in a 28 °C incubator to allow bacteria to grow. After one week, colonies with specific morphology (small colonies with white or pink color) were picked and purified on fresh MAG media with the addition of 2 mL NH_4_Cl solution (160 g/L). Purified strains were preserved at -80 °C in glycerol suspensions (30% v/v).

To identify the taxonomy of each strain, colony polymerase chain reaction (PCR) was performed. Briefly, bacterial colonies were mixed with the 10% (w/v) Chelex solution [Bio-Rad, USA] and incubated at 90 °C for 20 minutes to release DNA from bacterial colonies as templates. The universal bacteria 16S rRNA primers 27F (5′-AGRGTTYGATYMTGGCTCAG-3′) and 1492R (5′-GGYTACCTTGTTACGACTT-3′), Premix Taq [Takara Bio, USA], and RNA/DNAse-free water were used in the PCR amplification. The PCR condition included an initial denaturation at 95 °C for five minutes, followed by 35 cycles of amplification (95 °C for 45 s, 55 °C for 45 s, and 72 °C for 90 s) and a final extension at 72 °C for 10 minutes [3]. PCR products were run on 0.8% agarose and viewed by the automatic analysis system of electrophoresis gel imaging. Positive PCR products were sequenced in BGI Genomics. The taxonomic information of each isolate was analyzed by comparing the 16S rRNA gene sequences with *Bradyrhizobium* in EzBioCloud [4].

## Primer design for detecting *Bradyrhizobium* diversity

It is well known that the closely related genera are difficult to be distinguished by the traditional 16S rRNA gene sequences due to their slow evolutionary rate [5]. In contrast, the *rpoB* gene has a higher resolution and can be used as a marker gene to distinguish closely related species [5]. Therefore, we designed specific *Bradyrhizobium rpoB* primers for amplicon sequencing to investigate the relative abundance and diversity of *Bradyrhizobium* supergroups in different samples. The primer sequences were BR2106F (CCGRTSACGCCBGACAAG) and BR2516R (TGTCGCCCTTCYTGACGAYR), producing a ~410 bp sequence. The primers were validated to amplify various *Bradyrhizobium* members as well as strains within the PB supergroup with specificity (Fig. S12). PCR conditions included denaturation at 95 °C for three minutes, followed by 35 cycles of 95 °C for 30 seconds, 61 °C for 45 seconds, and 72 °C for 45 seconds, with a final extension at 72 °C for 10 minutes.

## Basic soil characteristics

Soil samples were separated to measure the basic soil characteristics after all the samples were brought back to the laboratory. Soil water content was determined by drying fresh soil (10.00 g) at 105°C for 6 hours. Soil pH was measured at the ratio of 1:5 (w/w) of soil-to-deionized water using a pH electrode. The potassium dichromate oxidation process combined with the heating method was used to measure the soil organic carbon (SOC). Total nitrogen (TN) was determined by the kjeldah method [6]. Total phosphorus (TP) was identified based on the sodium hydroxide melting-molybdenum antimony colorimetric method [6]. Available phosphorus (AP) was ascertained using the molybdenum antimony anti-colorimetric method [6]. Soil nitrite nitrogen (NO_2_^-^), nitrate nitrogen (NO_3_^-^), and ammonium nitrogen (NH_4_^+^) were extracted with KCl solution (2 mol L^-1^) and detected by continuous flow analysis [7]. Dissolved organic carbon and nitrogen (DOC and DON) were analyzed using a TOC/TN analyzer (Shimadzu, Analytical Sciences, Kyoto, Japan). Microbial biomass carbon and nitrogen (MBC and MBN) were determined by chloroform fumigation extraction [8, 9]. Fe and Mo contents were measured by using inductively coupled plasma mass spectrometry (ICP-MS). These sample-associated metadata were provided in Dataset S3.

## Amplicon sequence analysis

The quality and quantity of the DNA extracted from the soil and root samples were assessed using a NanoDropTM 2000 [Thermo Fisher, USA]. The amplicon sequence variants (ASVs) obtained from the *rpoB* amplicon sequences after denoising were subsequently filtered out the non-*rpoB* amplicon by conducting BLASTN searches against the NCBI nr database. The aligned ASVs with an e-value lower than 1e-10 but without alignment with ‘DNA-directed RNA polymerase subunit beta’ in the nr database were removed. The reference *rpoB* sequence was extracted from the genomic sequence and then aligned. The alignment was trimmed to retain the amplified region and used as the reference alignment. The ASVs were then aligned with the reference alignment using PaPaRa v2.5 [10]. The aligned ASVs were subsequently placed on the phylogenetic tree (Fig. S1B) using EPA-ng v0.3.8 [11] and assigned to seven *Bradyrhizobium* supergroups, including Soil 1, Soil 2, *Bradyrhizobium elkanii*, *Bradyrhizobium jicamae*, Kakadu, Photosynthetic *Bradyrhizobium*, and *Bradyrhizobium japonicum* supergroups. The relative abundance of each *Bradyrhizobium* supergroup was determined by dividing the number of reads assigned to each *Bradyrhizobium* supergroup by the total number of filtered reads assigned to *Bradyrhizobium* (Fig. 1A, Dataset S1). Similarly, the relative abundance of each clade of Photosynthetic *Bradyrhizobium* (PB) was calculated by dividing the number of reads assigned to each PB clade by the total number of filtered reads assigned to PB (Fig. 1B, Fig. S5, Dataset S2).

## Genome assembly and annotation

The BBMerge method in the BBmap package v38.79 was used to identify the untrimmed adapters associated with the raw reads [12]. Subsequently, Trimmomatic v0.39 was used to trim adapters and low-quality reads [13]. Reads with less than 40 bp in length were discarded, and the quality of the remaining reads was assessed by FastQC (<https://www.bioinformatics.babraham.ac.uk/projects/fastqc/>). Contigs were assembled using SPAdes v3.10.1 based on the remaining high-quality paired-end reads with default parameters [14]. Those contigs with lengths greater than 1,000 bp and a k-mer coverage greater than five were kept for further analysis. The quality of the genome assemblies (Dataset S8) was assessed by CheckM v1.0.7 [15]. In general, 122 newly sequenced strains were clustered with 12 publicly available strains as Clade 3, strains STM3843 and the newly sequenced HKCCYLS1011 were clustered as Clade 2, and the remaining 140 newly sequenced strains were clustered as Clade 1. Genomes from Clade 1 exhibited a significantly lower GC content (63.67 ± 0.07% vs. 65.46 ± 0.32%, *P* < 0.001, phylogenetic ANOVA) and a similar genome size (7.45 ± 0.14 Mb vs. 7.49 ± 0.36 Mb, *P* = 0.746, phylogenetic ANOVA) compared to Clade 3 (Datasets S7).

We noted an assembly error related to the *nif* island that was supposed to harbor two *nifH* genes. This was due to the presence of two *nifH* gene copies with nearly identical sequences, which can lead to mis-assembly in short-read sequencing technologies such as Illumina or MGISEQ-2000 [16]. To address this issue, we mapped all raw reads to the assembled genome and observed that the sequencing depth of the *nifH* gene was twice that of other nearby genes. We further conducted Nanopore sequencing on three phylogenetically distantly related PB strains (SZCCHNR3119, SZCCHNS2021, and SZCCHNS1050), and had their genomes assembled using Flye v2.6 [17]. Analysis of these genomes confirmed the presence of two *nifH* genes separated by approximately 43 genes in these strains.

To correct this error in the remaining PB strains, we re-assembled their genomes using the reference-guided method [18] in Spades v3.10.1 with the parameter of “--untrusted-contigs” [14]. For each assembled genome, genes were predicted using Prokka v1.12 [19] and annotated using the Cluster of Orthologous Genes (COG) database [20] and the Kyoto Encyclopedia of Genes and Genomes (KEGG) database [21, 22].

## Phylogenomic tree construction for all available *Bradyrhizobium* lineages

The parameter “-s alignment -spp partition -m MFP -mset LG,WAG,JTT -mrate E,G,I,G+I -bb 1000” was applied in the phylogenomic tree of all available *Bradyrhizobium* (Fig. S2) so that each gene was allowed to have its own best-fit substitution model, automatically selected by the ModelFinder implemented in IQ-TREE v2.2.0 [23]. The branch support was assessed by 1000 ultrafast bootstrap approximations [24].

## Phylogenomic and comparative genomic analyses for Photosynthetic *Bradyrhizobium*

OrthoFinder v2.3.4 [25] was used to identify orthologous gene families for all Photosynthetic *Bradyrhizobium* strains. Each of the identified 3,083 single-copy ortholog families was aligned at the amino acid level using MAFFT v7.487 [26], and trimmed using trimAl v1.4.rev15 with the parameters “-automated1 -resoverlap 0.55 - seqoverlap 60” [27]. A maximum likelihood phylogenomic tree of the PB supergroup was built based on the concatenated alignment of 3,083 single-copy orthologs using IQ-TREE v2.2.0 with 1,000 ultrafast bootstrap replicates [24]. The best-fit evolutionary model for each ortholog was determined by ModelFinder implemented in IQ-TREE v2.2.0 [23].

As shown in Fig. S2, the Kakadu supergroup was considered to be a sister lineage to the Photosynthetic supergroup. However, the long branches connecting these two supergroups suggested that using the Kakadu supergroup as an outgroup to root the species tree of the Photosynthetic supergroup may not be appropriate. Therefore, the Photosynthetic supergroup phylogenomic tree was rooted using outgroup-independent rooting methods, specifically the minimum variance (MV) [28] and the minimal ancestor deviation (MAD) [29] methods. The MV method identifies the root with the minimum variance of root-to-tip distances, while the MAD method considers all branches as plausible root positions, calculates the relative deviation from the clock-likeness for each candidate, and determines the root with the minimal mean relative deviation from the molecular clock interpretation of all branches [29]. The trees based on these two outgroup-independent methods indicated the same root position (Fig. 2 and Fig. S4). To compare continuous traits of strains while incorporating the evolution of those traits, a phylogenetic ANOVA [30] was conducted using the ‘phylANOVA’ function with 1,000 simulations implemented in the R package ‘phytools’ [31].

The similarity of the PB strains was measured at both 16S rRNA gene and whole-genome levels. For the former, pairwise 16S rRNA gene identity was calculated using BLAST [32], and strains were clustered using the complete linkage method based on sequence identity. For the latter, the whole-genome average nucleotide identity was estimated by FastANI v1.2 [33].

## Symbiotic analysis on *Aeschynomene indica*

The 28 strains used for symbiotic analysis were grown in AG medium [34] at 28°C on Petri dishes for one week. Bacteria were harvested from the plate and resuspended in 10 mL of sterile water and the OD_600_ was adjusted to 1.0. *A. indica* plants were cultured as previously described [35]. Each strain was inoculated into four plants with 1 mL of bacterial suspension and the symbiotic properties (number of nodules per plant and nitrogenase enzyme activity) were analyzed at 17 days after inoculation [36]. Cytological analysis of nodules elicited by one strain representative of the main clusters (MC2 to MC6), two strains for the Clade 2, and three strains for the MC1 was performed as described [37]. Each strain was assessed in duplicate.

## In vitro nitrogenase enzyme activity

Bacteria were grown in 9 mL vacuette® tubes (Greiner Bio-One GmbH) containing 2 mL of 0.8% agar BNM-B medium with 10 mM succinate and 10 mM arabinose as carbon source at 28 °C [38]. At the beginning of the experiment, 10% acetylene was added to the vacuette® tubes and after eight days of incubation, the amount of ethylene produced by the bacterial culture was measured by gas chromatography [39].

## Methanol metabolism

We selected 17 strains covering the main clusters predicted by PopCOGenT, along with the reference strain *Bradyrhizobium* diazoefficiens USDA110, which was used as a positive control, to perform the methanol metabolism assay. Each strain was inoculated into 14 mL test tubes containing 5 mL minimal salts medium, supplemented with 30 μM CeCl_2_ (lanthanide chlorides), and 0.5% methanol as the sole carbon source [40] and incubated at 28 ℃ with reciprocal shaking at 280 rpm. Growth was monitored spectrophotometrically by measuring the optical density at 600 nm (OD_600_).

# Supplementary references

1. Edwards J, Johnson C, Santos-Medellin C, Lurie E, Podishetty NK, Bhatnagar S, et al. Structure, variation, and assembly of the root-associated microbiomes of rice. *Proc Natl Acad Sci U S A* 2015; **112:** E911-920. https://doi.org/10.1073/pnas.1414592112

2. Mbai F, Magiri E, Matiru V, Nganga J, Nyambati V. Isolation and characterization of bacterial root endophytes with potential to enhance plant growth from Kenyan Basmati rice. *American International Journal of Contemporary Research* 2013; **3:** 25. https://repository.dkut.ac.ke:8080/xmlui/handle/123456789/7779

3. Tao J, Wang S, Liao T, Luo H. Evolutionary origin and ecological implication of a unique *nif* island in free-living *Bradyrhizobium* lineages. *ISME J* 2021; **15:** 3195-3206. https://doi.org/10.1038/s41396-021-01002-z

4. Yoon SH, Ha SM, Kwon S, Lim J, Kim Y, Seo H, et al. Introducing EzBioCloud: a taxonomically united database of 16S rRNA gene sequences and whole-genome assemblies. *Int J Syst Evol Microbiol* 2017; **67:** 1613-1617. https://doi.org/10.1099/ijsem.0.001755

5. Vos M, Quince C, Pijl AS, de Hollander M, Kowalchuk GA. A comparison of *rpoB* and 16S rRNA as markers in pyrosequencing studies of bacterial diversity. *PLOS ONE* 2012; **7:** e30600. https://doi.org/10.1371/journal.pone.0030600

6. Bao S (2000). Soil agrochemical analysis. China agriculture press Beijing.

7. Tian XF, Hu HW, Ding Q, Song MH, Xu XL, Zheng Y, et al. Influence of nitrogen fertilization on soil ammonia oxidizer and denitrifier abundance, microbial biomass, and enzyme activities in an alpine meadow. *Biology and Fertility of Soils* 2014; **50:** 703-713. https://doi.org/10.1007/s00374-013-0889-0

8. Durenkamp M, Luo Y, Brookes PC. Impact of black carbon addition to soil on the determination of soil microbial biomass by fumigation extraction. *Soil Biology & Biochemistry* 2010; **42:** 2026-2029. https://doi.org/10.1016/j.soilbio.2010.07.016

9. Wu J, Joergensen RG, Pommerening B, Chaussod R, Brookes PC. Measurement of soil microbial biomass C by fumigation extraction - an automated procedure. *Soil Biology & Biochemistry* 1990; **22:** 1167-1169. https://doi.org/10.1016/0038-0717(90)90046-3

10. Berger SA, Stamatakis A. PaPaRa 2.0: a vectorized algorithm for probabilistic phylogeny-aware alignment extension. *Heidelberg Institute for Theoretical Studies* 2012; **12**.

11. Barbera P, Kozlov AM, Czech L, Morel B, Darriba D, Flouri T, et al. EPA-ng: massively parallel evolutionary placement of genetic sequences. *Syst Biol* 2019; **68:** 365-369. https://doi.org/10.1093/sysbio/syy054

12. Bushnell B, Rood J, Singer E. BBMerge–accurate paired shotgun read merging via overlap. *PLOS ONE* 2017; **12:** e0185056. https://doi.org/10.1371/journal.pone.0185056

13. Bolger AM, Lohse M, Usadel B. Trimmomatic: a flexible trimmer for Illumina sequence data. *Bioinformatics* 2014; **30:** 2114-2120. https://doi.org/10.1093/bioinformatics/btu170

14. Bankevich A, Nurk S, Antipov D, Gurevich AA, Dvorkin M, Kulikov AS, et al. SPAdes: a new genome assembly algorithm and its applications to single-cell sequencing. *J Comput Biol* 2012; **19:** 455-477. 10.1089/cmb.2012.0021

15. Parks DH, Imelfort M, Skennerton CT, Hugenholtz P, Tyson GW. CheckM: assessing the quality of microbial genomes recovered from isolates, single cells, and metagenomes. *Genome Res.* 2015; **25:** 1043-1055. http://www.genome.org/cgi/doi/10.1101/gr.186072.114

16. Tørresen OK, Star B, Mier P, Andrade-Navarro MA, Bateman A, Jarnot P, et al. Tandem repeats lead to sequence assembly errors and impose multi-level challenges for genome and protein databases. *Nucleic Acids Research* 2019; **47:** 10994-11006. https://doi.org/10.1093/nar/gkz841

17. Kolmogorov M, Yuan J, Lin Y, Pevzner PA. Assembly of long, error-prone reads using repeat graphs. *Nat Biotechnol* 2019; **37:** 540-546. https://doi.org/10.1038/s41587-019-0072-8

18. Cabuk U, Unlu ES. A combined *de novo* assembly approach increases the quality of prokaryotic draft genomes. *Folia Microbiol (Praha)* 2022; **67:** 801-810. https://doi.org/10.1007/s12223-022-00980-7

19. Seemann T. Prokka: rapid prokaryotic genome annotation. *Bioinformatics* 2014; **30:** 2068-2069. <https://doi.org/10.1093/bioinformatics/btu153>

20. Galperin MY, Wolf YI, Makarova KS, Vera Alvarez R, Landsman D, Koonin EV. COG database update: focus on microbial diversity, model organisms, and widespread pathogens. *Nucleic Acids Research* 2020; **49:** D274-D281. https://doi.org/10.1093/nar/gkaa1018

21. Kanehisa M, Furumichi M, Sato Y, Kawashima M, Ishiguro-Watanabe M. KEGG for taxonomy-based analysis of pathways and genomes. *Nucleic Acids Res* 2023; **51:** D587-D592. https://doi.org/10.1093/nar/gkac963

22. Kanehisa M, Goto S, Kawashima S, Okuno Y, Hattori M. The KEGG resource for deciphering the genome. *Nucleic Acids Res* 2004; **32:** D277-280. https://doi.org/10.1093/nar/gkh063

23. Minh BQ, Schmidt HA, Chernomor O, Schrempf D, Woodhams MD, von Haeseler A, et al. IQ-TREE 2: new models and efficient methods for phylogenetic inference in the genomic era. *Molecular Biology and Evolution* 2020; **37:** 1530-1534. <https://doi.org/10.1093/molbev/msaa015>

24. Hoang DT, Chernomor O, von Haeseler A, Minh BQ, Vinh LS. UFBoot2: improving the ultrafast bootstrap approximation. *Molecular Biology and Evolution* 2017; **35:** 518-522. https://doi.org/10.1093/molbev/msx281

25. Emms DM, Kelly S. OrthoFinder: phylogenetic orthology inference for comparative genomics. *Genome Biol* 2019; **20:** 238. https://doi.org/10.1186/s13059-019-1832-y

26. Katoh K, Standley DM. MAFFT multiple sequence alignment software version 7: improvements in performance and usability. *Mol Biol Evol* 2013; **30:** 772-780. https://doi.org/10.1093/molbev/mst010

27. Capella-Gutiérrez S, Silla-Martínez JM, Gabaldón T. trimAl: a tool for automated alignment trimming in large-scale phylogenetic analyses. *Bioinformatics* 2009; **25:** 1972-1973. https://doi.org/10.1093/bioinformatics/btp348

28. Mai U, Sayyari E, Mirarab S. Minimum variance rooting of phylogenetic trees and implications for species tree reconstruction. *PLOS ONE* 2017; **12:** e0182238. https://doi.org/10.1371/journal.pone.0182238

29. Tria FDK, Landan G, Dagan T. Phylogenetic rooting using minimal ancestor deviation. *Nat Ecol Evol* 2017; **1:** 193. https://doi.org/10.1038/s41559-017-0193

30. Garland T, Dickerman AW, Janis CM, Jones JA. Phylogenetic analysis of covariance by computer-simulation. *Systematic Biology* 1993; **42:** 265-292. https://doi.org/10.1093/sysbio/42.3.265

31. Revell LJ. phytools 2.0: An updated R ecosystem for phylogenetic comparative methods (and other things). 2024; **12:** e16505. https://doi.org/10.7717/peerj.16505

32. Boratyn GM, Camacho C, Cooper PS, Coulouris G, Fong A, Ma N, et al. BLAST: a more efficient report with usability improvements. *Nucleic Acids Res* 2013; **41:** W29-33. https://doi.org/10.1093/nar/gkt282

33. Jain C, Rodriguez RL, Phillippy AM, Konstantinidis KT, Aluru S. High throughput ANI analysis of 90K prokaryotic genomes reveals clear species boundaries. *Nat Commun* 2018; **9:** 5114. https://doi.org/10.1038/s41467-018-07641-9

34. Sadowsky MJ, Tully RE, Cregan PB, Keyser HH. Genetic diversity in *Bradyrhizobium* *japonicum* serogroup 123 and its relation to genotype-specific nodulation of soybean. *Appl Environ Microbiol* 1987; **53:** 2624-2630. https://doi.org/10.1128/aem.53.11.2624-2630.1987

35. Okazaki S, Tittabutr P, Teulet A, Thouin J, Fardoux J, Chaintreuil C, et al. Rhizobium-legume symbiosis in the absence of *Nod* factors: two possible scenarios with or without the T3SS. *ISME J* 2016; **10:** 64-74. https://doi.org/10.1038/ismej.2015.103

36. Bonaldi K, Gourion B, Fardoux J, Hannibal L, Cartieaux F, Boursot M, et al. Large-scale transposon mutagenesis of photosynthetic *Bradyrhizobium* sp. strain ORS278 reveals new genetic loci putatively important for *nod*-independent symbiosis with *Aeschynomene indica*. *Mol Plant Microbe Interact* 2010; **23:** 760-770. https://doi.org/10.1094/MPMI-23-6-0760

37. Songwattana P, Chaintreuil C, Wongdee J, Teulet A, Mbaye M, Piromyou P, et al. Identification of type III effectors modulating the symbiotic properties of *Bradyrhizobium vignae* strain ORS3257 with various *Vigna* species. *Sci Rep* 2021; **11:** 4874. https://doi.org/10.1038/s41598-021-84205-w

38. Nouwen N, Arrighi J-F, Cartieaux F, Chaintreuil C, Gully D, Klopp C, et al. The role of rhizobial (NifV) and plant (FEN1) homocitrate synthases in *Aeschynomene*/photosynthetic *Bradyrhizobium* symbiosis. *Sci Rep* 2017; **7:** 448. https://doi.org/10.1038/s41598-017-00559-0

39. Giraud E, Hannibal L, Fardoux J, Vermeglio A, Dreyfus B. Effect of *Bradyrhizobium* photosynthesis on stem nodulation of *Aeschynomene sensitiva*. *Proc Natl Acad Sci U S A* 2000; **97:** 14795-14800. https://doi.org/10.1073/pnas.250484097

40. Wang L, Suganuma S, Hibino A, Mitsui R, Tani A, Matsumoto T, et al. Lanthanide-dependent methanol dehydrogenase from the legume symbiotic nitrogen-fixing bacterium *Bradyrhizobium diazoefficiens* strain USDA110. *Enzyme Microb Technol* 2019; **130:** 109371. https://doi.org/10.1016/j.enzmictec.2019.109371
